# Dalpulapans A–E from the roots of *Dalbergia stipulacea*†

**DOI:** 10.1039/d1ra07041j

**Published:** 2021-11-23

**Authors:** Priyapan Posri, Thurdpong Sribuhom, Sookkawath Walunchapruk, Thanaset Senawong, Sarawut Tontapha, Vittaya Amornkitbamrung, Chavi Yenjai

**Affiliations:** Natural Products Research Unit, Center of Excellence for Innovation in Chemistry, Department of Chemistry, Faculty of Science, Khon Kaen University Khon Kaen 40002 Thailand chayen@kku.ac.th +66-4320-2222-41 ext. 12243; Natural Products Research Unit, Department of Biochemistry, Faculty of Science, Khon Kaen University Khon Kaen 40002 Thailand; Integrated Nanotechnology Research Centre, Department of Physics, Faculty of Science, Khon Kaen University Khon Kaen 40002 Thailand

## Abstract

Five new compounds, dalpulapans A–E (1–5), were isolated from the hexane extract of the roots of *Dalbergia stipulacea* Roxb. Five new compounds, dalpulapans A–E (1–5), were isolated from the hexane extract of the roots of *Dalbergia stipulacea* Roxb. An evaluation of cytotoxic activity against HeLa, A549 and normal cell lines using MTT assay was performed. The results showed that *R*,*R*-velucarpin A (6) was the most active against HeLa cells with an IC_50_ value of 10.9 ± 0.42 μM, while fortunately this compound exhibited weak cytotoxicity against normal cells (29.20 ± 1.16 μM). Structures of all isolates were identified from their 1D and 2D NMR spectroscopic data and MS analysis. Experimental and calculated ECD spectra were studied to define the absolute configurations.

## Abstract 2

Five new compounds, dalpulapans A–E (1–5), were isolated from the hexane extract of the roots of *Dalbergia stipulacea* Roxb.

## Graphical abstract

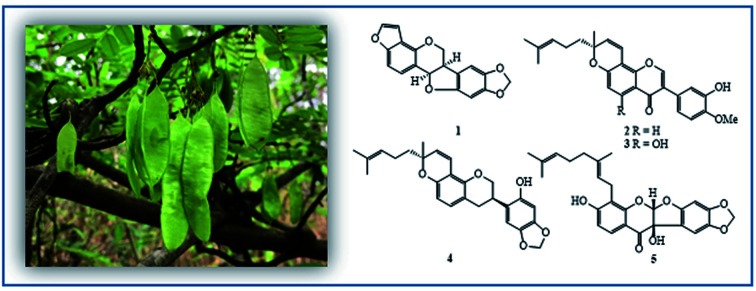

## 1. Introduction

*Dalbergia stipulacea* Roxb., which belongs to the family Fabaceae, is found throughout southern China, eastern India, Myanmar, Thailand, Vietnam and Laos and is known as “Ma Kham Tao” in Thai. This plant has been used as an emmenagogue and for abortion when taken in moderate amounts. It is believed that this plant can be used for gonorrhea, syphilis and mouth ulcers.^1^ Moreover, the roots of this plant are poisonous to fish.^2^ The chemical constituents from this plant which include isoflavonoids, chalcone, pterocarpan and phenylpropene have been reported.^2,3^ Antifungal activity against *Pythium insidiosum*, a fungus-like microorganism for which at present there is no effective agent for treatment, was evaluated and shows interesting results.^4^ Further investigation of compounds from the roots of this plant and testing for cytotoxicity against A549 (lung cancer cells), HeLa (cervical cancer cells) and Vero cells (kidney of African green monkey cells; normal cells) was made. In this work, five new compounds (dalpulapans 1–5) and seven known compounds were reported. The absolute configurations of chiral carbons were determined using experimental electronic circular dichroism (ECD) analysis and comparing the specific rotations with those previously reported.

## 2. Discussion

**Fig. 1:**
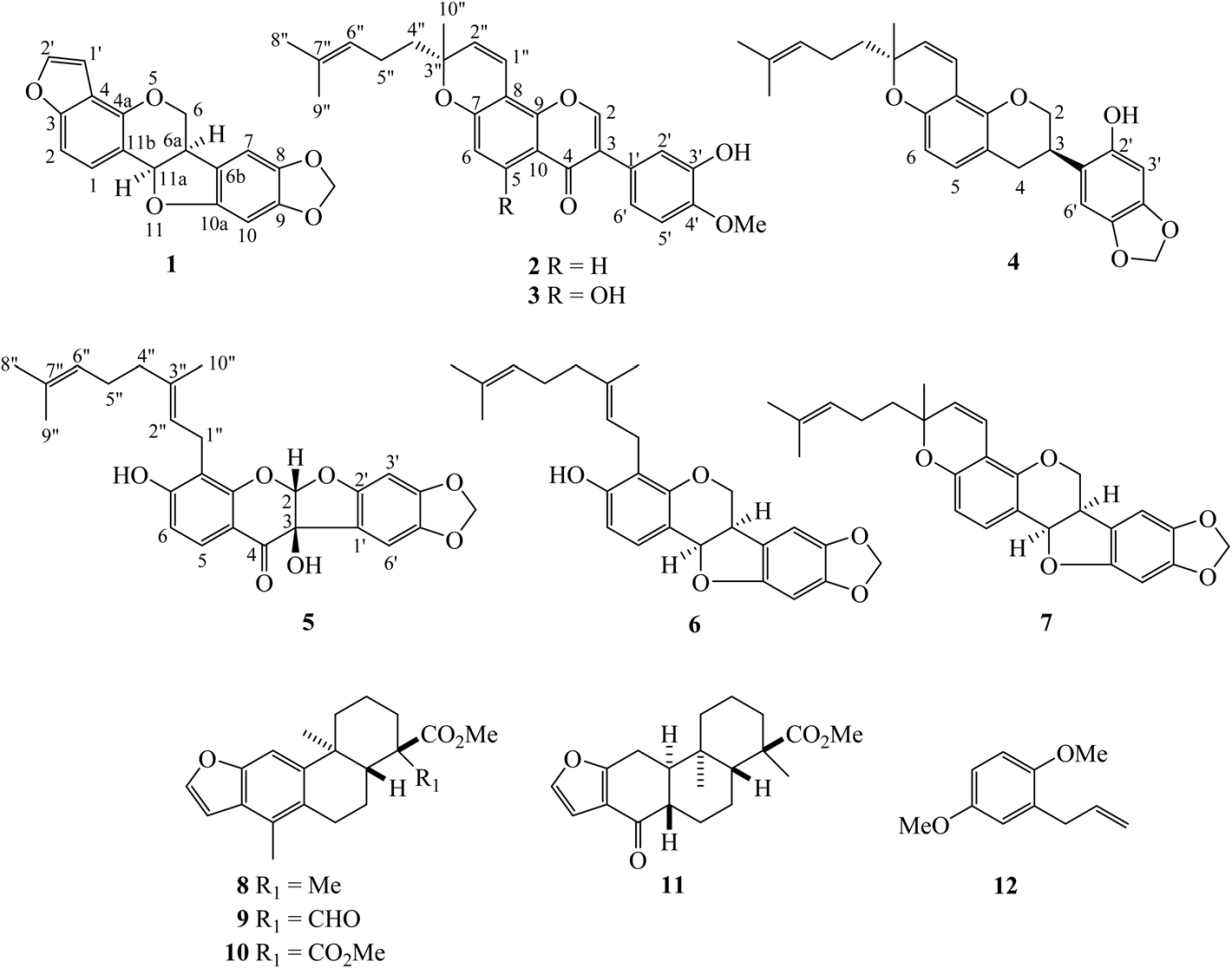
Structures of all isolated compounds 1–12.

The extraction and isolation of hexane extract of the roots of *D. stipulacea* by chromatographic methods led to five new compounds named dalpulapans A–E (1–5) (Fig. 1) and seven known compounds (6–12) including *R*,*R*-velucarpin A (6),^5^*R*,*R*-velucarpin C (7),^5,6^ taepeenin A (8),^7^ taepeenin E (9),^7^ pteroloterin A (10),^8^ nortaepeenin A (11)^8^ and 2-allyl-1,4-dimethoxybenzene (12) (Fig. 1). All chemical structures were identified by spectroscopic methods, HRESIMS and ECD data.

**Fig. 2:**
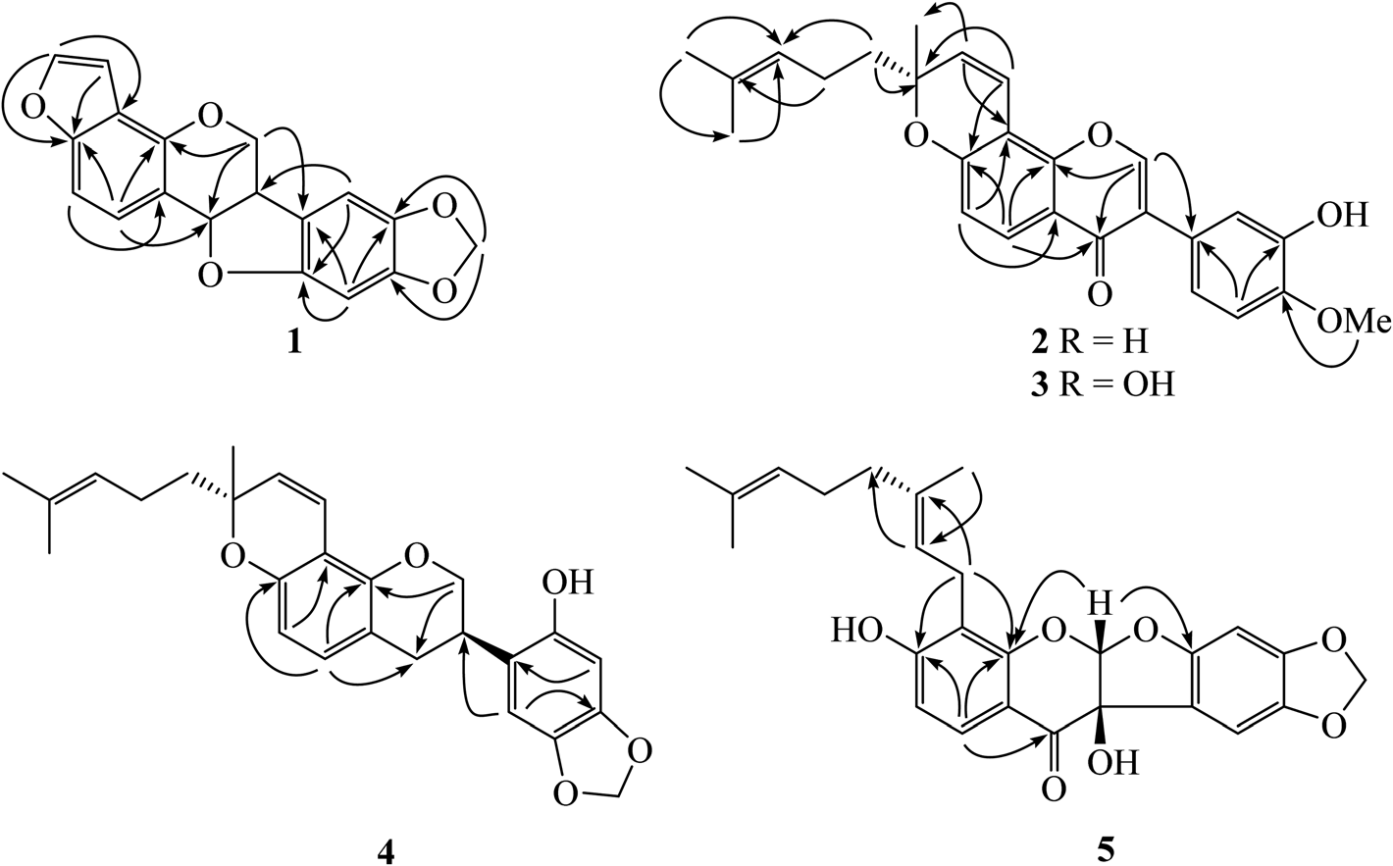
Key HMBC correlations of compounds 1–5.

Compound 1 showed the molecular formula C_18_H_12_O_5_ identified from its HRESIMS ion at *m*/*z* 331.0580 [M + Na]^+^ (calcd 331.0582). It contains five oxygenated aromatic carbons at *δ*_C_ 156.4 (C-3), 154.4 (C-10a), 149.6 (C-4a), 148.3 (C-9) and 141.9 (C-8) from the ^13^C NMR data (Table 1). The ^1^H NMR displayed two doublet signals (*J* = 8.4 Hz) at *δ*_H_ 7.42 and *δ*_H_ 7.22 of aromatic protons H-1 and H-2, respectively. This molecule contains a furan moiety, shown by proton signals at *δ*_H_ 6.85 (*J* = 2.0 Hz, H-1′) and *δ*_H_ 7.56 (*J* = 2.0 Hz, H-2′); in addition, these protons were linked to carbons at *δ*_C_ 144.5 and *δ*_C_ 104.2, respectively. The HMBC correlations between H-1 (*δ*_H_ 7.42) and C-3 (*δ*_C_ 156.4), C-4a (*δ*_C_ 149.6) and C-11a (*δ*_C_ 78.8), and between H-2 (*δ*_H_ 7.22) and C-4 (*δ*_C_ 117.4) and C-11b (*δ*_C_ 113.1), and between H-2′ (*δ*_H_ 7.56) and C-3 (*δ*_C_ 156.4) and C-4 (*δ*_C_ 117.4) confirmed the connectivity of a furan moiety at the C-3 and C-4 positions (Fig. 2). Two singlet signals of aromatic protons H-7 (*δ*_H_ 6.86) and H-10 (*δ*_H_ 6.46) were evident. Methylenedioxy protons were observed at *δ*_H_ 5.90 and *δ*_H_ 5.92 in the ^1^H NMR spectrum and connected to the same carbon at *δ*_C_ 101.6. A doublet of doublets signal at *δ*_H_ 4.37 (*J* = 10.8, 4.8 Hz) was assigned to H-6α, while a triplet signal at *δ*_H_ 3.76 (*J* = 10.8 Hz) was given as H-6β. The coupling constant of oxymethine proton H-11a was 7.2 Hz, confirming the cofacial orientation to H-6a.^9^ Long-range couplings between H-7 (*δ*_H_ 6.86) and C-6a (*δ*_C_ 40.3) and C-10a (*δ*_C_ 154.4), and between H-10 (*δ*_H_ 6.46) and C-6b (*δ*_C_ 118.1) and C-10a (*δ*_C_ 154.4) were detected in the HMBC spectrum (Fig. 2). The absolute configuration was confirmed by comparison with the ECD spectrum and specific rotation with pterocarpan (Chem. Rev. 2013).^10^ The compound 1 showed a negative optical rotation value ([*α*]^27^_D_ −104.7) and displayed negative Cotton effect at 238 nm (Δ*ε* −41.14). Kaennakam and coworkers reported the specific rotation of velucarpin A as [*α*]^20^_D_ −83.8 and also showed negative Cotton effect at 247 (Δ*ε* −8.71).^5^ Thus, the absolute configuration of compound 1 was 6a*R*,11a*R* and it was named dalpulapan A.

**Table 1:** ^1^H and ^13^C NMR data for compounds 1–5 in CDCl_3_ (*δ* in ppm)

Position	1		Position	2		3		4		5	
	*δ*_H_ (*J* in Hz)	*δ*_C_		*δ*_H_ (*J* in Hz)	*δ*_C_	*δ*_H_ (*J* in Hz)	*δ*_C_	*δ*_H_ (*J* in Hz)	*δ*_C_	*δ*_H_ (*J* in Hz)	*δ*_C_
1	7.42, d (8.4)	126.9	1	—	—	—	—	—	—	—	
2	7.22, d (8.4)	105.9	2	7.93, s	152.0	7.87, s	152.6	4.33, dd (10.4, 1.4)	70.1	6.33, s	110.3
3		156.4		—		—		3.99, t (10.4)		—	
4		117.4	3	—	124.8	—	123.6	3.51, m	32.0	—	80.4
4a		149.6	4	—	175.9	—	180.9	2.92, dd (15.7, 10.3)	30.8	—	189.4
6α	4.37, dd (10.8, 4.8)	66.7		—		—		2.83, dd (15.7, 5.2)		—	
6β	3.76, t (10.8)		5	8.05, d (9.0)	126.9	—	162.4	6.81, d (8.2)	129.3	7.62, d (8.8)	127.1
6a	3.55, m	40.3	6	6.84, d (9.0)	115.1	6.28, s	100.2	6.36, d (8.2)	108.8	6.55, d (8.8)	112.2
6b		118.1	7	—	157.7	—	160.0	—	152.3	—	162.9
7	6.86, s	104.9	8	—	109.1	—	101.0	—	109.9	—	117.9
8		141.9	9	—	152.4	—	152.2	—	149.8	—	158.1
9		148.3	10	—	118.4	—	106.0	—	114.0	—	115.3
10	6.46, s	94.0	1′	—	125.3	—	124.0	—	128.1	—	110.3
10a		154.4	2′	7.11, s	115.6	7.07, d (2.0)	115.3	—	148.0	—	155.6
11a	5.61, d (7.2)	78.8	3′	—	145.7	—	145.8	6.37, s	98.5	6.48, s	94.2
11b		113.1	4′	—	146.7	—	147.0	—	146.6	—	150.5
1′	6.85, d (2.0)	144.5	5′	6.92, d (8.0)	110.8	6.91, d (8.0)	110.9	—	142.0	—	143.4
2′	7.56, d (2.0)	104.2	6′	7.11, d (8.0)	121.3	7.05, dd (2.0,8.0)	121.1	6.60, s	107.1	6.66, s	103.7
OCH_2_O	5.92, d (1.2)	101.6	1′′	6.84, d (10.0)	115.3	6.71, d (10.2)	115.2	6.67, d (10.0)	117.4	3.46, d (6.8)	22.3
	5.90, d (1.2)										
			2′′	5.66, d (10.0)	129.4	5.53, d (10.2)	126.4	5.51, d (10.0)	128.1	5.23, t (6.8)	120.3
			3′′	—	80.3	—	80.7	—	78.1	—	140.3
			4′′	1.77, m	41.6	1.73, m	41.7	1.71, m	41.0	2.08, m	39.8
			5′′	2.12, m	22.8	2.10, m	22.7	2.10, m	22.8	2.08, m	26.4
			6′′	5.10, t (6.8)	123.8	5.09, t (7.2)	123.8	5.09, t (7.0)	124.4	5.04, t (6.8)	123.7
			7′′	—	132.2	—	132.1	—	131.7	—	132.3
			8′′	1.66, s	25.8	1.66, s	25.8	1.66, s	25.8	1.66, s	25.8
			9′′	1.57, s	17.8	1.58, s	17.8	1.58, s	17.8	1.59, s	17.9
			10′′	1.46, s	27.0	1.44, s	27.1	1.39, s	26.3	1.81	16.4
			OCH_2_O	—	—	—	—	5.89, d (1.2)	101.3	5.92, d (1.2),	102.0
								5.88, d (1.2)		5.89, d (1.2)	
			–OH	5.65, s	—	5.71, s	—	4.74, s	—	4.16, s	—
						12.95, s				6.16, s	
			–OCH_3_	3.92, s	56.2	3.91, s	56.2	—	—	—	—

Isoflavone derivative 2, dalpulapan B, showed the molecular formula of C_26_H_26_O_5_ identified from its HRESIMS ion at *m*/*z* 419.1858 [M + H]^+^ (calcd for C_26_H_27_O_5_, 419.1858). The singlet signal at *δ*_H_ 7.93 was located at oxygenated carbon C-2 (*δ*_C_ 152.0); in addition, this proton correlated with C-3 (*δ*_C_ 124.8), C-4 (*δ*_C_ 175.9) and C-9 (*δ*_C_ 152.4) in the HMBC spectrum, indicating the isoflavone core structure. The protons H-5 (*δ*_H_ 8.05) and H-6 (*δ*_H_ 6.84) showed two doublet signals with a coupling constant of 9.0 Hz (Table 1). The HMBC correlations were found between H-5 (*δ*_H_ 8.05) and C-4 (*δ*_C_ 175.9), C-7 (*δ*_C_ 157.7) and C-9 (152.4). The protons on the B-ring displayed an ABX system at *δ*_H_ 7.11 (d, *J* = 8.0 Hz, H-6′), *δ*_H_ 6.92 (d, *J* = 8.0 Hz, H-5′) and *δ*_H_ 7.11 (s, H-2′). Cross-peaks between H-5′ (*δ*_H_ 6.92) to C-1′ (*δ*_C_ 125.3) and C-3′ (*δ*_C_ 145.7) and between methoxy proton (*δ*_H_ 3.92) and C-4′ (*δ*_C_ 146.7) were observed in the HMBC data. The presence of a pyran ring and prenyl group were detected in the ^1^H and ^13^C NMR spectra. The olefinic protons in the pyran ring resonated at *δ*_H_/*δ*_C_ 6.84/115.3 of 1′′ position and at *δ*_H_/*δ*_C_ 5.66/129.4 of 2′′ position, in addition, an oxygenated quaternary carbon, C-3′′ exhibited at *δ*_C_ 80.3. The ^1^H NMR data displayed a triplet signal of an olefinic proton H-6′′ (*δ*_H_ 5.10, t, *J* = 6.8 Hz) which coupled with H-5′′ (*δ*_H_ 2.12, m). Cross-peaks between CH_3_-8′′ and CH_3_-9′′ and C-6′′ were observed in the HMBC data. The experimental ECD spectrum displayed negative Cotton effects at 233 nm (Δ*ε* −11.50) and positive Cotton effects at 265 nm (Δ*ε* +7.89) which was similar to the calculated spectrum for the (3′*R*) configuration confirming the structure of 2 as shown in Fig. 1.

The ^1^H and ^13^C NMR data from dalpulapan C (3) were similar to 2, except for the presence of a hydroxy group at the C-5 position in compound 3. The molecular formula, C_26_H_26_O_6_, confirmed the additional oxygen atom compared to 2. An aromatic proton H-6 (*δ*_H_ 6.28, s) showed long-range coupling with C-5 (*δ*_C_ 162.4), C-7 (*δ*_C_ 160.0), C-8 (*δ*_C_ 101.0) and C-10 (*δ*_C_ 106.0) in the HMBC experiment (Table 1). Intramolecular H-bonding was detected at *δ*_H_ 12.95, confirming the presence of a hydroxy group at the C-5 position, in addition, this hydroxy proton showed long-range coupling with C-5, C-6 and C-10 in the HMBC data. The specific rotation of compound 3, [*α*]^28^_D_ +142.5, was the same as compound 2 and the experimental ECD was match to calculated ECD of 3*R*′ configuration. Thus the structure of compound 3 was identified as shown in Fig. 1.

Dalpulapan D (4) possessed a protonated adduct ion at *m*/*z* 421.2006 corresponding to the molecular formula C_26_H_28_O_5_. This molecule was an isoflavan derivative and contained a pyran moiety, as characterized from 1D and 2D NMR data. An oxygenated methylene proton at *δ*_H_ 4.33 (dd, *J* = 10.4, 1.4 Hz, H-2a) and *δ*_H_ 3.99 (t, *J* = 10.4 Hz, H-2b) correlated with carbon at *δ*_C_ 70.1 in the HMQC data. From the multiplicity and coupling constant, it can be identified that H-2b was located at the axial position. Two doublet of doublet signals at *δ*_H_ 2.92 (*J* = 15.7, 10.3 Hz, H-4a) and *δ*_H_ 2.83 (*J* = 15.7, 5.2 Hz, H-4b) were located on the carbon at *δ*_C_ 30.8. The multiplet signal of a methine proton H-3 showed at *δ*_H_ 3.51. The connection of the H-2/H-3/H-4 system was observed in the ^1^H–^1^H COSY data. Two singlet signals at *δ*_H_ 6.37 and *δ*_H_ 6.60 were assigned as H-3′ and H-6′, respectively. This compound showed a methylenedioxy group at *δ*_H_ 5.89 and *δ*_H_ 5.88 and located on the same carbon at *δ*_C_ 101.3. Cross-peaks between H-6′ (*δ*_H_ 6.60) and C-3 (*δ*_C_ 32.0) and between H-3′ (*δ*_H_ 6.37) and C-1′ (*δ*_C_ 128.1) were observed in the HMBC spectrum. The ^1^H and ^13^C NMR spectra displayed the containing of pyran ring and prenyl side chain as compounds 2 and 3. Compound 4 showed positive Cotton effect at 218 nm (Δ*ε* +2.28) and 284 nm (Δ*ε* +5.75) and a negative positive Cotton effect at 243 nm (Δ*ε* −3.32), which corresponded with the calculated ECD spectrum of 3*R*,3′′*R* configuration.^11,12^ It should be note that the absolute configuration at C-3′′ was the same as compounds 2 and 3. All data concluded that the structure of 4 was shown in Fig. 1.

Dalpulapan E (5) was given a molecular formula C_26_H_26_O_7_ characterized from the negative molecular ion peak [M − H]^+^ at *m*/*z* 449.1598. The signals of the aromatic protons H-5 and H-6 displayed as doublets (*J* = 8.8 Hz) at *δ*_H_ 7.62 and *δ*_H_ 6.55 and correlated with carbons at *δ*_C_ 127.1 and *δ*_C_ 112.2, respectively, in the HMQC experiment. Two singlet signals on the aromatic B-ring were evident (*δ*_H_ 6.48, H-3′ and *δ*_H_ 6.66, H-6′). Methylenedioxy protons showed two doublets (*J* = 1.2 Hz) at *δ*_H_ 5.92 and *δ*_H_ 5.89 and were located at carbon *δ*_C_ 102.0. This compound exhibited a geranyl group by showing two olefinic protons at *δ*_H_ 5.23 (t, *J* = 6.8 Hz, H-2′′) and *δ*_H_ 5.04 (t, *J* = 6.8 Hz, H-6′′), three methylene protons and three methyl protons. The HMBC cross-peaks showed correlation between H-1′′ (*δ*_H_ 3.46) and oxygenated carbons C-7 (*δ*_C_ 162.9) and C-9 (*δ*_C_ 158.1), which confirmed the hydroxy group at the C-7 position. A singlet signal proton at *δ*_H_ 6.33 (H-2) was located at C-2 (*δ*_C_ 110.3), which bears two oxygen atoms. This proton correlated with carbons C-9 (*δ*_C_ 158.1) and C-2′ (*δ*_C_ 155.6) in the HMBC spectrum, maintaining the presence of an acetal group. The broad singlet signal at *δ*_H_ 4.16 was assigned as a hydroxy proton, OH-3, and in addition the ^13^C NMR signal of the oxygenated quaternary carbon C-3 exhibited at *δ*_C_ 80.4. The experimental ECD data, shows a negative Cotton effect at 239 nm (Δ*ε* −32.31) and a positive Cotton effect at 315 nm (Δ*ε* +13.47) and possessed positive specific rotation at [*α*]^27.5^_D_ +141.3. These information corresponded with the calculated ECD spectrum of 2*S*,3*R* configuration. In addition, both ECD data and the specific rotation of 5 are opposite to previous report, (2*R*,3*S*)-3,7,4′-trihydroxy-5-methoxycoumaronochromone.^13^ That compound showed negative specific rotation at [*α*]^22^_D_ −184.6; and positive and negative Cotton effects at 211 nm (Δ*ε* +19.9) and 292 nm (Δ*ε* −19.9), respectively. Thus the absolute configuration at C-2 and C-3 were confirmed as 2*S*,3*R* as shown in Fig. 1.

All isolated compounds, except 12, were evaluated for cytotoxicity against A549 (lung cancer cells), HeLa (cervical cancer cells) and Vero cells using the MTT assay. The cytotoxicity results showed the most active compound was 6, which exhibited an IC_50_ value of 10.9 ± 0.42 μM against HeLa cells. In addition, IC_50_ values against A549 and Vero cells were 14.6 ± 1.31 and 29.2 ± 1.16 μM, respectively. The remaining compounds showed inactive (IC_50_ > 15 μM) to the test.

## 3. Experimental section

### 3.1. General experimental procedures

A Sanyo Gallenkamp (UK) melting point apparatus was used to find the melting points. Optical rotations were measured on a JASCO P-1020 digital polarimeter (Japan). The UV spectra were obtained on an Agilent 8453 UV-visible spectrophotometer (Germany). A PerkinElmer Spectrum One FT-IR spectrophotometer (USA) was used to acquire the IR spectra. NMR spectra were collected at 400 MHz (^1^H) and at 100 MHz (^13^C) using a Varian Mercury Plus spectrometer (USA). HRESIMS was performed on a Micromass Q-TOF 2 hybrid quadrupole time-of-flight (Q-TOF) mass spectrometer (Micromass, UK). Analytical thin-layer chromatography (TLC) was accomplished on Merck silica gel 60 F_254_. Column chromatography separations were carried out on silica gel less than 0.063 mm, 0.063–0.200 mm or RP-18.

### 3.2. Plant material

The roots of *D. stipulacea* were collected in February 2018 at Ban Na Phaeng Village, Wiang Kao District, Khon Kaen Province, Thailand (16°41′53.5′′N, 102°20′24.1′′E). Plant material (voucher specimen KKU012018) was identified by Assoc. Prof. Suppachai Tiyaworanant, Faculty of Pharmaceutical Sciences, Khon Kaen University, Thailand.

### 3.3. Extraction and isolation

The dried powdered roots (12 kg) of *D. stipulacea* were extracted with hexanes (15 L × 3), EtOAc (10 L × 3) and MeOH (10 L × 3) at ambient temperature. The solutions were concentrated *in vacuo* to give the crude hexane (162 g, 1.35%), EtOAc (368 g, 3.07%) and crude MeOH (649 g, 5.41%) extracts. The crude hexane extract (162 g) was subjected to column chromatography (silica gel 60) and obtained five fractions, F1–F5. Fraction F2 was purified by FCC (hexane : EtOAc) to give 7 (10.35 g) and 8 (0.12 g). Fraction F3 was further purified and gave four fractions, F3.1–F3.4. Compound 1 (7.5 mg) was obtained from F3.1 while compounds 4 (5.7 mg) and 12 (3.2 mg) were found from F3.2. Subfraction F3.4 was chromatographed on a silica gel column (90 : 10 hexane : EtOAc) to obtain two subfractions, F3.4.1 and F3.4.2. Compounds 9 (5.5 mg) and 10 (9.9 mg) were found from F3.4.2. Fraction F4 was chromatographed out on silica gel FCC, eluting with hexane : EtOAc (70 : 30) to obtain four subfractions, F4.1–F4.4. Compounds 3 (59.1 mg), 6 (0.40 g) and 11 (36.6 mg) were obtained from subfraction F4.1. Compound 5 (7.0 mg) was obtained from F4.2. Subfraction F4.4 was purified and gave 2 (3.2 mg).

#### Dalpulapan A (1)

A brown solid; mp 191–194 °C; [*α*]^27^_D_ −104.7 (*c* 0.1, CHCl_3_); UV (CH_3_OH) *λ*_max_ (log *ε*) 249 (4.39), 311 (4.13) nm; ECD 205 nm (Δ*ε* +37.91), 215 nm (Δ*ε* −102.05); IR (neat) *ν*_max_ 2884, 1602, 1476, 1372, 1142, 1057, 935, 766 cm^−1^; ^1^H NMR (400 MHz) and ^13^C NMR (100 MHz) data (CDCl_3_), see Table 1; HRESIMS *m*/*z* 331.0580 [M + Na]^+^ (calcd for C_18_H_12_O_5_Na, 331.0582).

#### Dalpulapan B (2)

A yellowish oil; [*α*]^28^_D_ +144.0 (*c* 0.1, CHCl_3_); UV (CH_3_OH) *λ*_max_ (log *ε*) 265 (4.80) nm; IR (neat) *ν*_max_ 3367, 2929, 1626, 1576, 1511, 1438, 1395, 1274, 1196, 1134, 1080, 906, 807, 762 cm^−1^; ^1^H NMR (400 MHz) and ^13^C NMR (100 MHz) data (CDCl_3_), see Table 1; HRESIMS *m*/*z* 419.1858 [M + H]^+^ (calcd for C_26_H_27_O_5_, 419.1858).

#### Dalpulapan C (3)

Yellowish solid; mp 121–124 °C; [*α*]^28^_D_ +142.5 (*c* 0.1, CHCl_3_); UV (CH_3_OH) *λ*_max_ (log *ε*) 274 (4.38) nm; IR (neat) *ν*_max_ 3442, 2926, 1615, 1574, 1512, 1429, 1376, 1274, 1174, 1081, 1033, 954, 913, 819, 764 cm^−1^; ^1^H NMR (400 MHz) and ^13^C NMR (100 MHz) data (CDCl_3_), see Table 1; HRESIMS *m*/*z* 435.1808 [M + H]^+^ (calcd for C_26_H_27_O_6_, 435.1808).

#### Dalpulapan D (4)

An brown oil; [*α*]^28^_D_ +1.9 (*c* 0.1, CHCl_3_); UV (CH_3_OH) *λ*_max_ (log *ε*) 209 (4.05), 282 (3.51) nm; IR (neat) *ν*_max_ 3728, 3404, 2921, 1634, 1610, 1586, 1479, 1440, 1378, 1280, 1213, 1168, 1096, 1064, 1038, 935, 863, 772, 720 cm^−1^; ^1^H NMR (400 MHz) and ^13^C NMR (100 MHz) data (CDCl_3_), see Table 1; HRESIMS *m*/*z* 421.2006 [M + H]^+^ (calcd for C_26_H_29_O_5_, 421.2015).

#### Dalpulapan E (5)

A yellowish oil; [*α*]^27.5^_D_ +141.3 (*c* 0.1, CHCl_3_); UV (CH_3_OH) *λ*_max_ (log *ε*) 204 (4.58), 298 (4.06) nm; IR (neat) *ν*_max_ 3352, 2923, 1663, 1598, 1466, 1445, 1280, 1216, 1148, 1034, 992, 938, 826 cm^−1^; ^1^H NMR (400 MHz) and ^13^C NMR (100 MHz) data (CDCl_3_), see Table 1; HRESIMS *m*/*z* 449.1598 [M − H]^+^ (calcd for C_26_H_25_O_7_, 449.1600).

### 3.4. Cytotoxic activity assay

MTT (3-(4,5-dimethylthiazol-2-yl)-2,5-diphenyltetrazolium bromide) assay was performed to evaluate the cytotoxic effect of the compounds. The procedure has been explained in a previous report.^14^ In brief, all cells were cultured in RPMI 1640 medium supplemented with 10% fetal bovine serum, penicillin (100 U mL^−1^) and streptomycin (100 μg mL^−1^) (Gibco-BRL, USA) and incubated at 37 °C in a humidified atmosphere of 5% CO_2_. For preliminary testing, cells were exposed to the selected compounds at a concentration of 100 μg mL^−1^ for 24, 48 and 72 hours. The compounds that caused less than 50% cell viability were further evaluated for their half maximal inhibitory concentration (IC_50_) values. Control groups were treated with a solvent (a mixture of DMSO and ethanol; 1 : 1). After incubation, the medium was replaced with 110 μL of fresh medium containing MTT (0.5 mg mL^−1^ in PBS) (Sigma Chemical Co., St. Louis, MO, USA) and incubated for 2 h. The formazan formed after conversion of MTT was dissolved in DMSO. The absorbance of formazan was measured with a microplate reader (Bio-Rad Laboratories, USA) at the wavelength of 550 nm using 655 nm as a reference wavelength. Each assay was replicated four times. The percentage of viable cells which corresponds to the production of formazan was calculated as previously described (Kumnerdkhonkaen *et al.* 2018).% Cell viability = [sample (A550 − A655)/control (A550 − A655)] × 100

### 3.5. Calculation

The preliminary conformational analyses were evaluated using HyperChem software. These dominant conformers were further optimized at the B3LYP/6-311g (d,p) basis set by density functional theory.^15^ The GAUSSIAN 09 program was used to calculate the ECD spectra.^16^ The single point energy calculations were computed using time-dependent density functional theory (TD–DFT)^17^ at the CAM–B3LYP/6-311++g (d,p) level of theory.^18^ The CPCM polarizable conductor calculation model was used for bulk solvent effects.^19^

## 4. Conclusions

In this study, twelve compounds were isolated from hexane extract of the roots of *Dalbergia stipulacea* Roxb. They were five new compounds, dalpulapans A–E (1–5), and seven known compounds. The cytotoxicity evaluation against HeLa, A549 and normal cell lines using MTT assay was examined. It was found that 6 was the most active against HeLa cells with IC_50_ value of 10.9 ± 0.42 μM, and showed weak cytotoxicity against normal cells (29.20 ± 1.16 μM). The structures of all compounds were determined by 1D and 2D NMR spectroscopic studies. MS analysis and experimental and calculated ECD spectra were also studied to define the absolute configurations of new compounds.

## Conflicts of interest

The authors declare no competing financial interests.

## Supplementary Material

RA-011-D1RA07041J-s001
