# Supplementary material for: Dalpulapans A–E from the roots of Dalbergia stipulacea
Source: RSC Adv. 2021 Nov 23;11(59):37643–8. doi: 10.1039/d1ra07041j (PMC9043826; doi:10.1039/d1ra07041j)
Supplement: RA-011-D1RA07041J-s001 [file RA-011-D1RA07041J-s001.pdf]

Electronic Supplementary Material (ESI) for RSC Advances.
This journal is © The Royal Society of Chemistry 2021

Supporting Information

Dalpulapans A-E from the Roots of *Dalbergia stipulacea*

Priyapan Posri,[a] Thurdpong Sribuhom,[a] Sookkawath Walunchapruk,[b] Thanaset Senawong,[b] Sarawut Tontapha,[c] Vittaya Amornkitbamrung,[c] and Chavi Yenjai*[a]

[a]Natural Products Research Unit, Center of Excellence for Innovation in Chemistry, Department of Chemistry, Faculty of Science, Khon Kaen University, Khon Kaen 40002, Thailand

[b]Natural Products Research Unit, Department of Biochemistry, Faculty of Science, Khon Kaen University, Khon Kaen 40002, Thailand.

[c]Integrated Nanotechnology Research Centre, Department of Physics, Faculty of Science, Khon Kaen University, Khon Kaen 40002, Thailand

Authors to whom correspondence should be addressed.

*Tel: +66-4320-2222-41 ext. 12243. Fax: +66-4320-2373. E-mail: chayen@kku.ac.th (C. Yenjai).

Contents

Figure S1. ^{1}H NMR spectrum of dalpulapan A (**1**)

Figure S2. ^{13}C NMR spectrum of dalpulapan A (**1**)

Figure S3. ^{1}H-^{1}H COSY spectrum of dalpulapan A (**1**)

Figure S4. HMQC spectrum of dalpulapan A (**1**)

Figure S5. HMBC spectrum of dalpulapan A (**1**)

Figure S6. MS spectrum of dalpulapan A (**1**)

Figure S7. ECD spectrum of dalpulapan A (**1**)

Figure S8. ^{1}H NMR spectrum of dalpulapan B (**2**)

Figure S9. ^{13}C NMR spectrum of dalpulapan B (**2**)

Figure S10. ^{1}H-^{1}H COSY spectrum of dalpulapan B (**2**)

Figure S11. HMQC spectrum of dalpulapan B (**2**)

Figure S12. HMBC spectrum of dalpulapan B (**2**)

Figure S13. MS spectrum of dalpulapan B (**2**)

Figure S14. ECD spectrum of dalpulapan B (**2**)

Figure S15. ^{1}H NMR spectrum of dalpulapan C (**3**)

Figure S16. ^{13}C NMR spectrum of dalpulapan C (**3**)

Figure S17. ^{1}H-^{1}H COSY spectrum of dalpulapan C (**3**)

Figure S18. HMQC spectrum of dalpulapan C (**3**)

Figure S19. HMBC spectrum of dalpulapan C (**3**)

Figure S20. MS spectrum of dalpulapan C (**3**)

Figure S21. ECD spectrum of dalpulapan B (**2**)

Figure S22. ^{1}H NMR spectrum of dalpulapan D (**4**)

Figure S23. ^{13}C NMR spectrum of dalpulapan D (**4**)

Figure S24. ^{1}H-^{1}H COSY spectrum of dalpulapan D (**4**)

Figure S25. HMQC spectrum of dalpulapan D (**4**)

Figure S26. HMBC spectrum of dalpulapan D (**4**)

Figure S27. MS spectrum of dalpulapan D (**4**)

Figure S28. ECD spectrum of dalpulapan B (**4**)

Figure S29. ^{1}H NMR spectrum of dalpulapan E (**5**)

Figure S30. ^{13}C NMR spectrum of dalpulapan E (**5**)

Figure S31. ^{1}H-^{1}H COSY spectrum of dalpulapan E (**5**)

Figure S32. HMQC spectrum of dalpulapan E (**5**)

Figure S33. HMBC spectrum of dalpulapan E (**5**)

Figure S34. MS spectrum of dalpulapan E (**5**)

Figure S35. Experimental and calculated ECD spectrum of (2*S*,3*R*) dalpulapan E (**5**)

Figure S36. Calculated ECD spectrum of (2*R*,3*R* and 2*S*,3*S*) derivatives of **5**

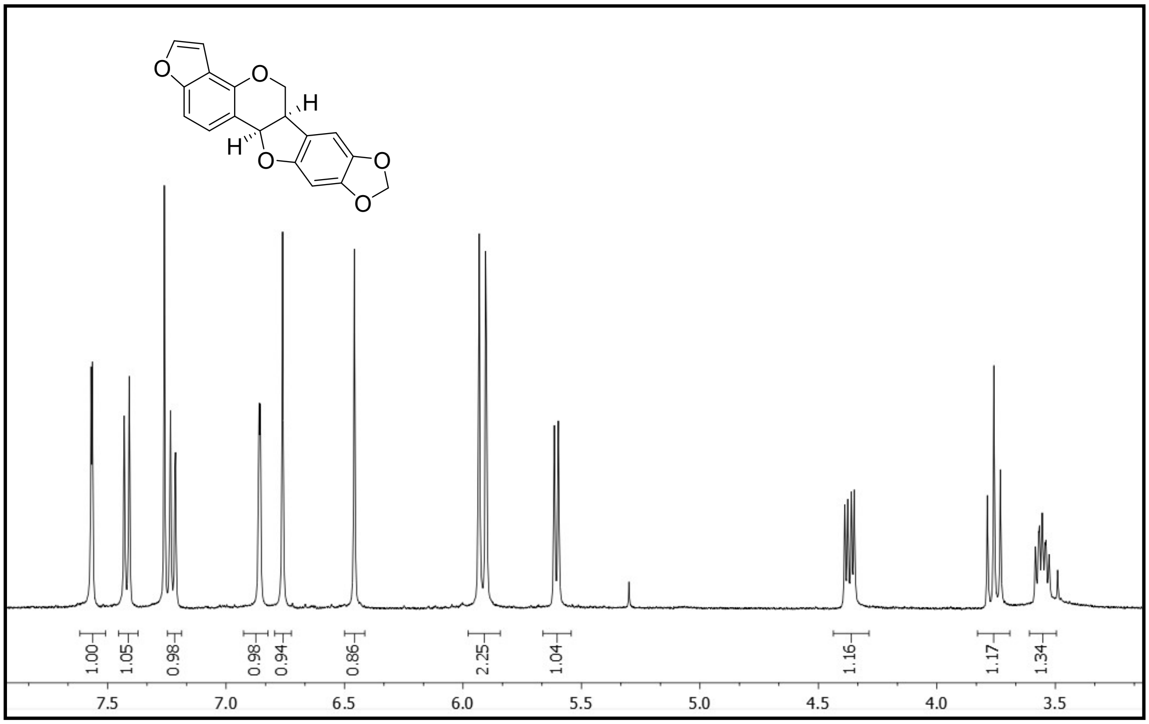

Figure S1. ^{1}H NMR spectrum of dalpulapan A (**1**)

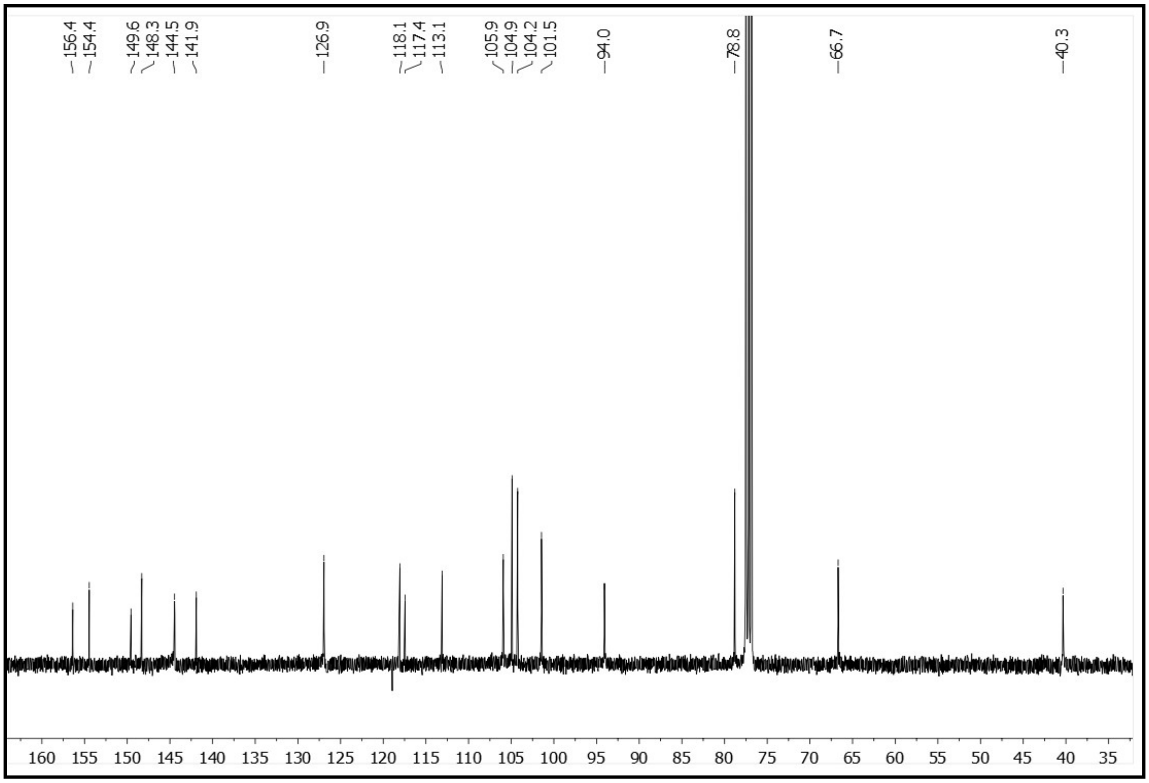

Figure S2. ^{13}C NMR spectrum of dalpulapan A (**1**)

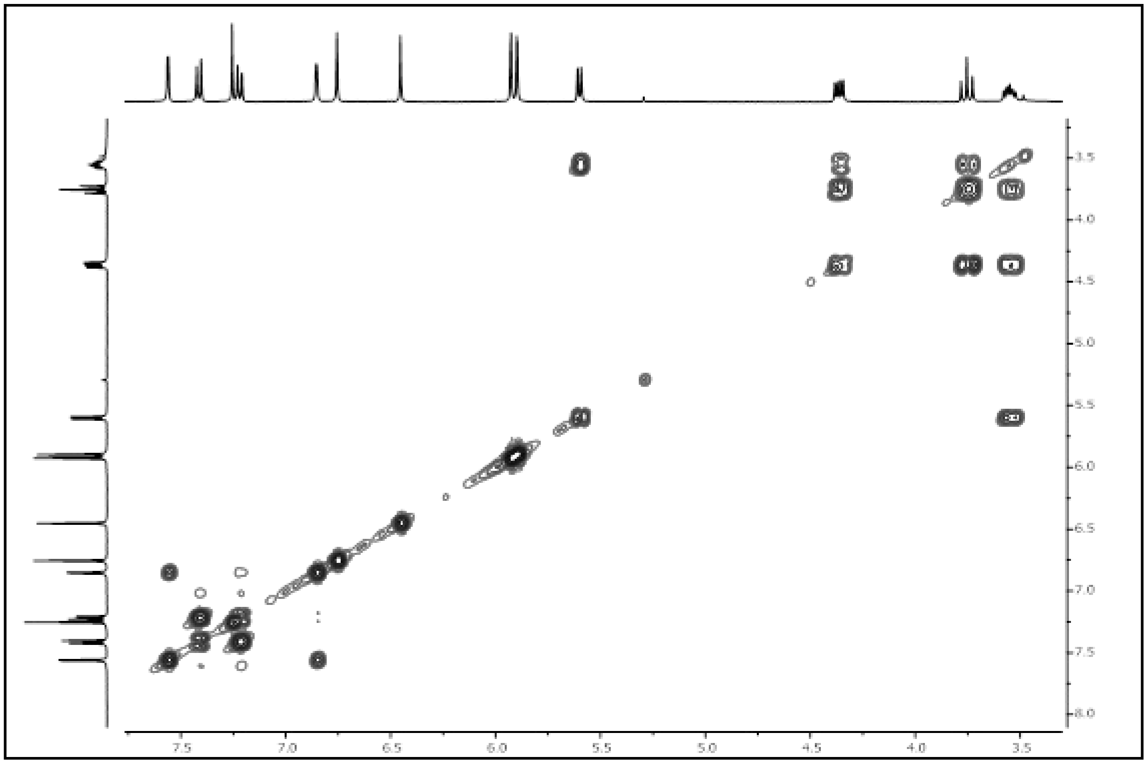

Figure S3. ¹H-¹H COSY spectrum of dalpulapan A (**1**)

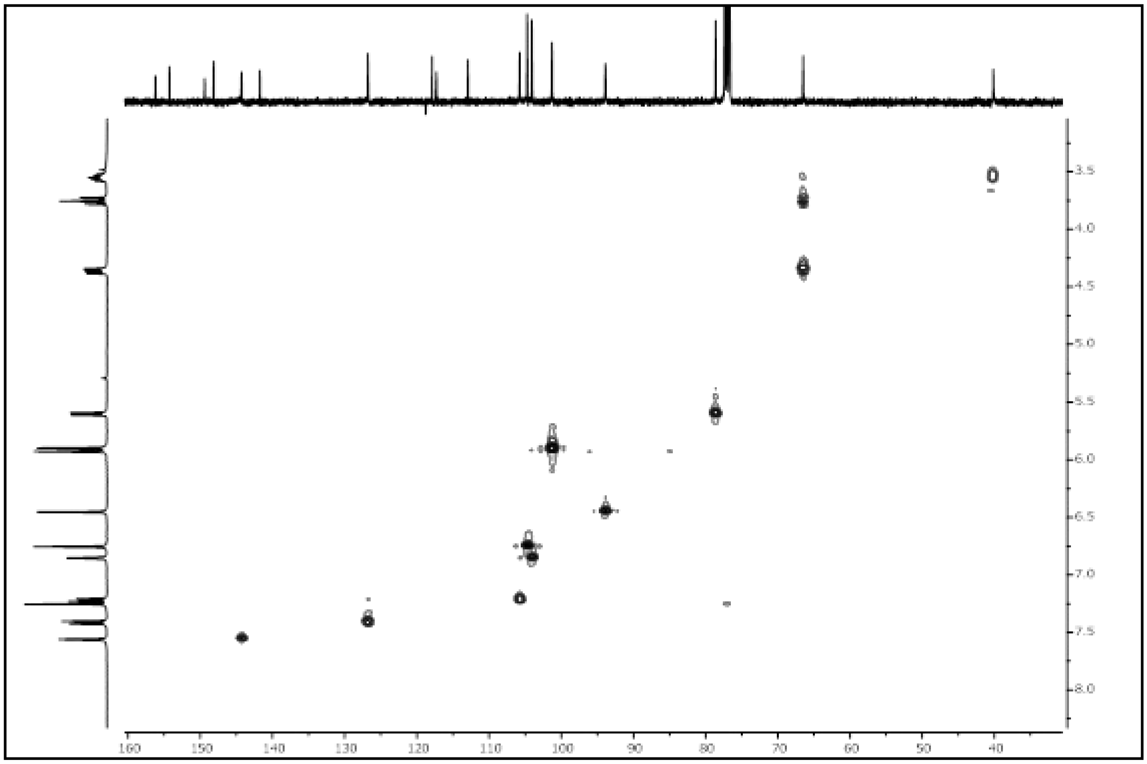

Figure S4. HMQC spectrum of dalpulapan A (**1**)

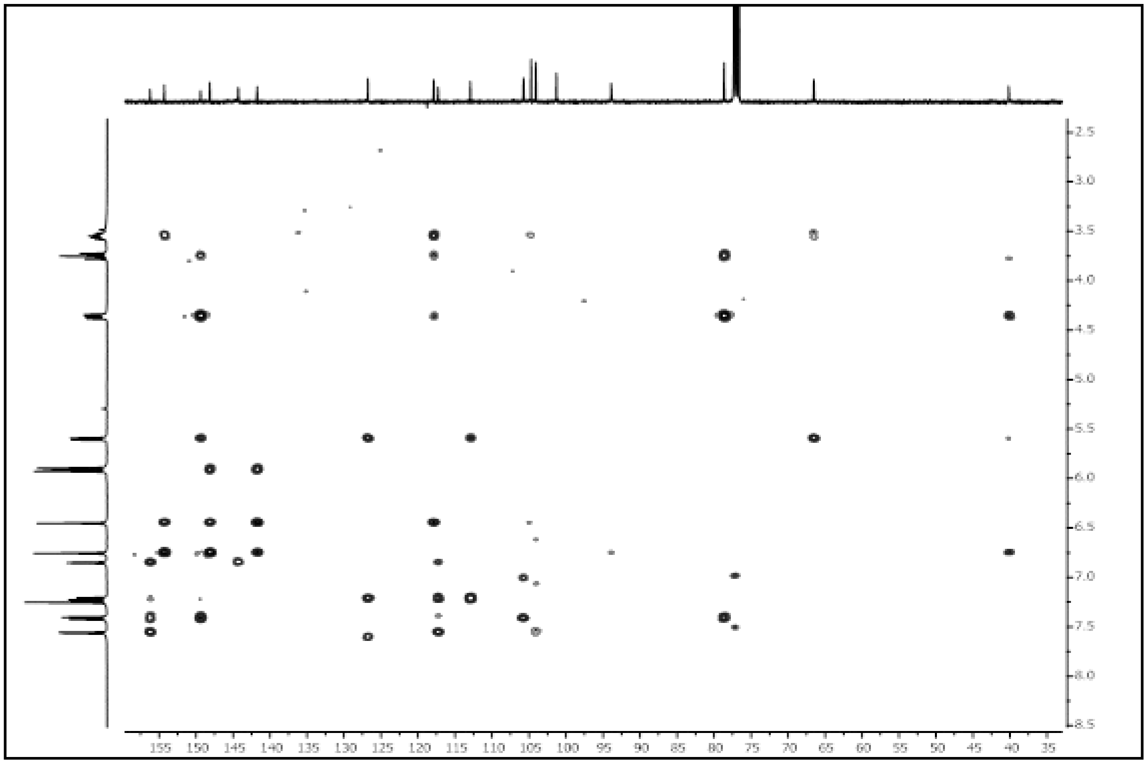

Figure S5. HMBC spectrum of dalpulapan A (**1**)

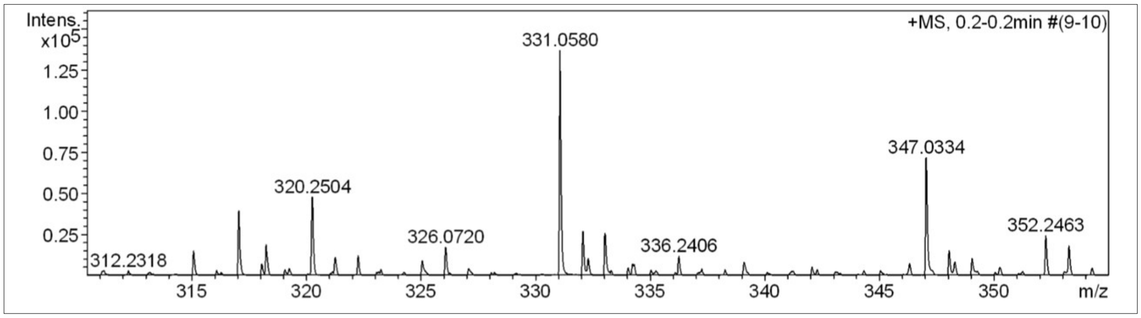

Figure S6. MS spectrum of dalpulapans A (**1**)

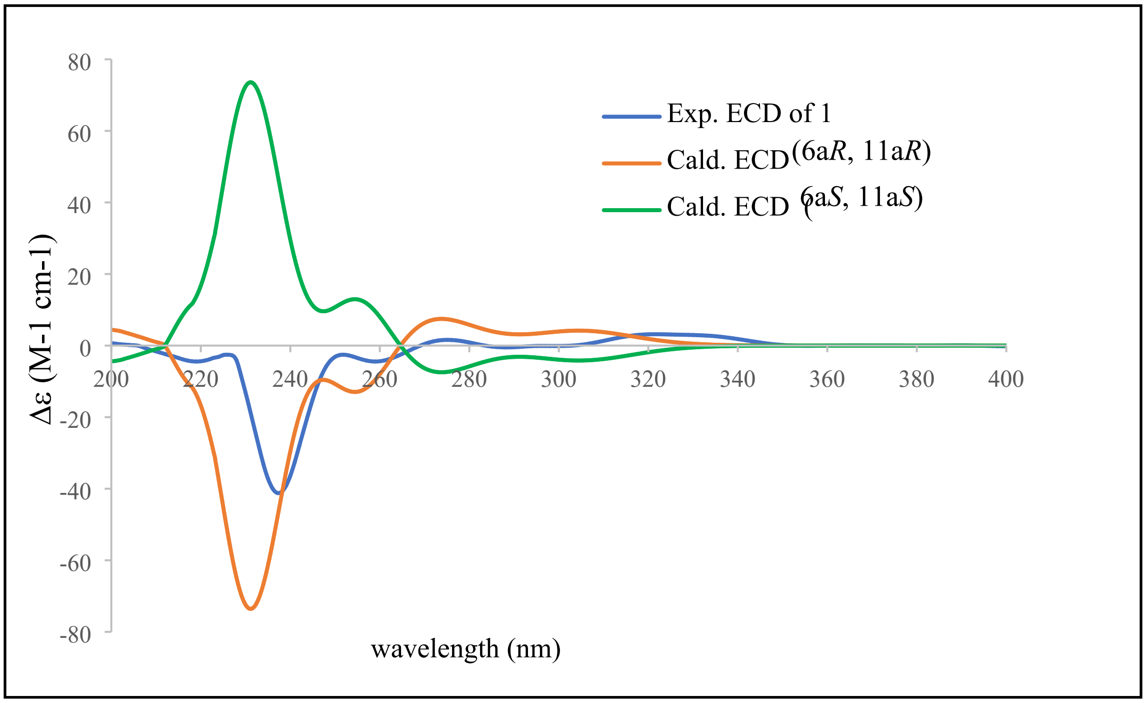

Figure S7. ECD spectrum of dalpulapan A (**1**)

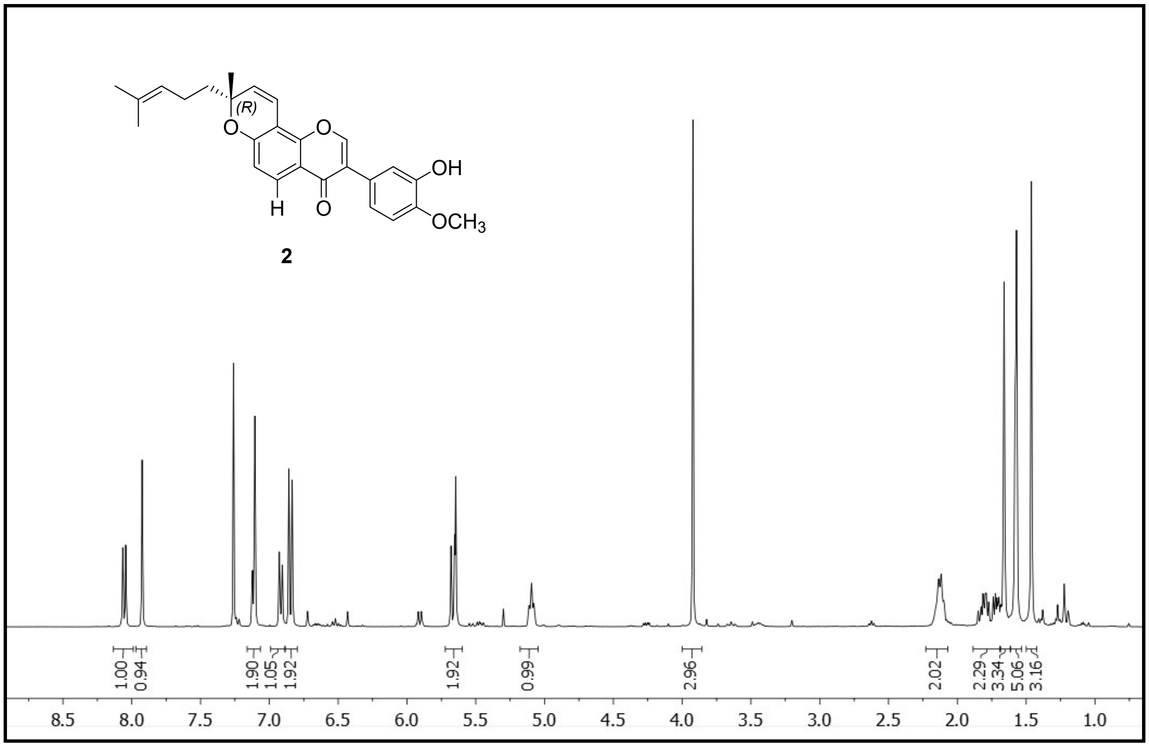

Figure S8. ^{1}H NMR spectrum of dalpulapan B (**2**)

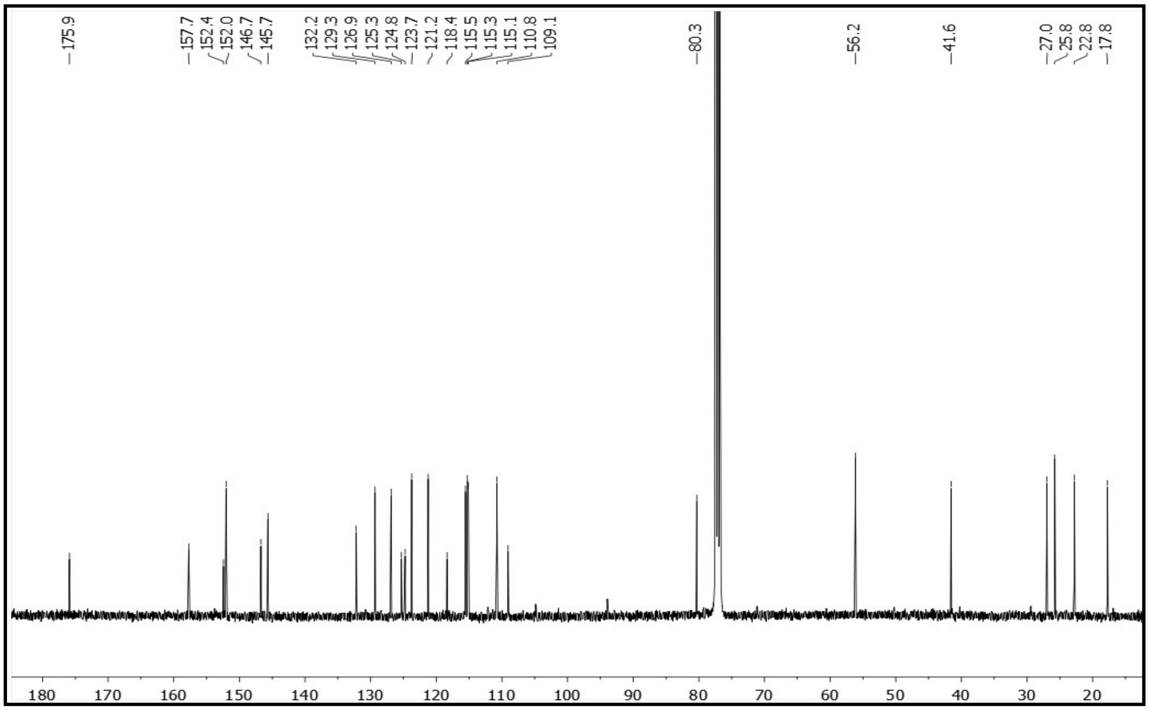

Figure S9. ^{13}C NMR spectrum of dalpulapan B (**2**)

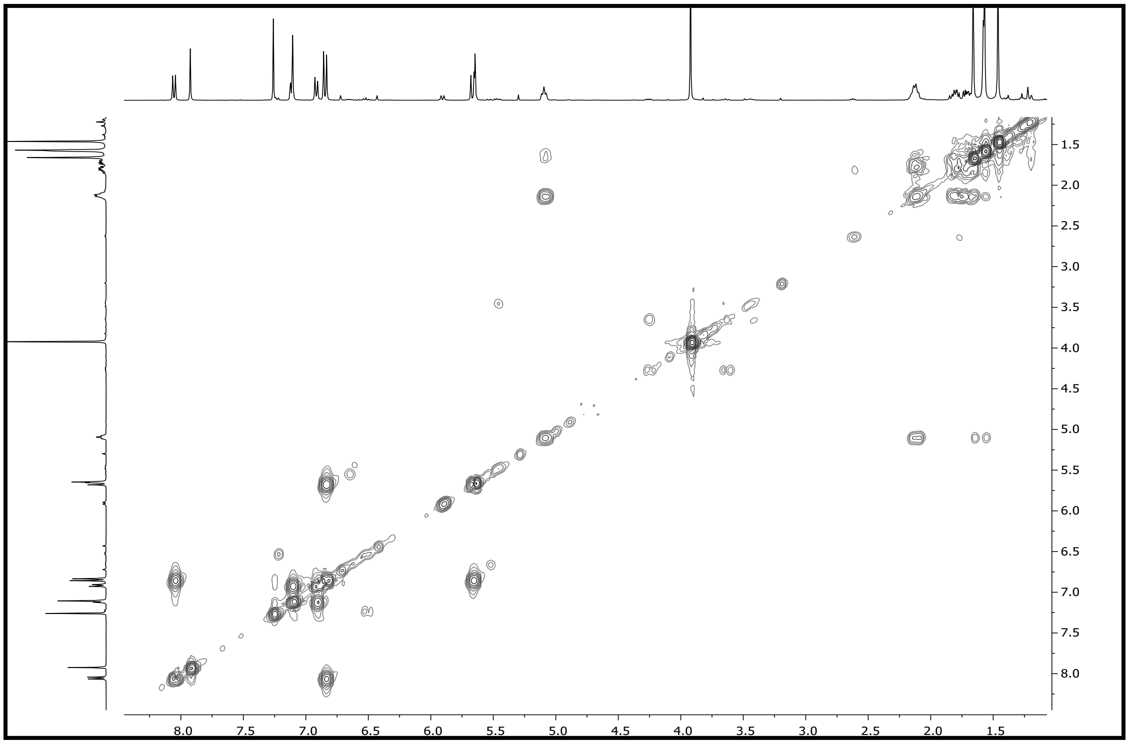

Figure S10. ^{1}H-^{1}H COSY spectrum of dalpulapan B (**2**)

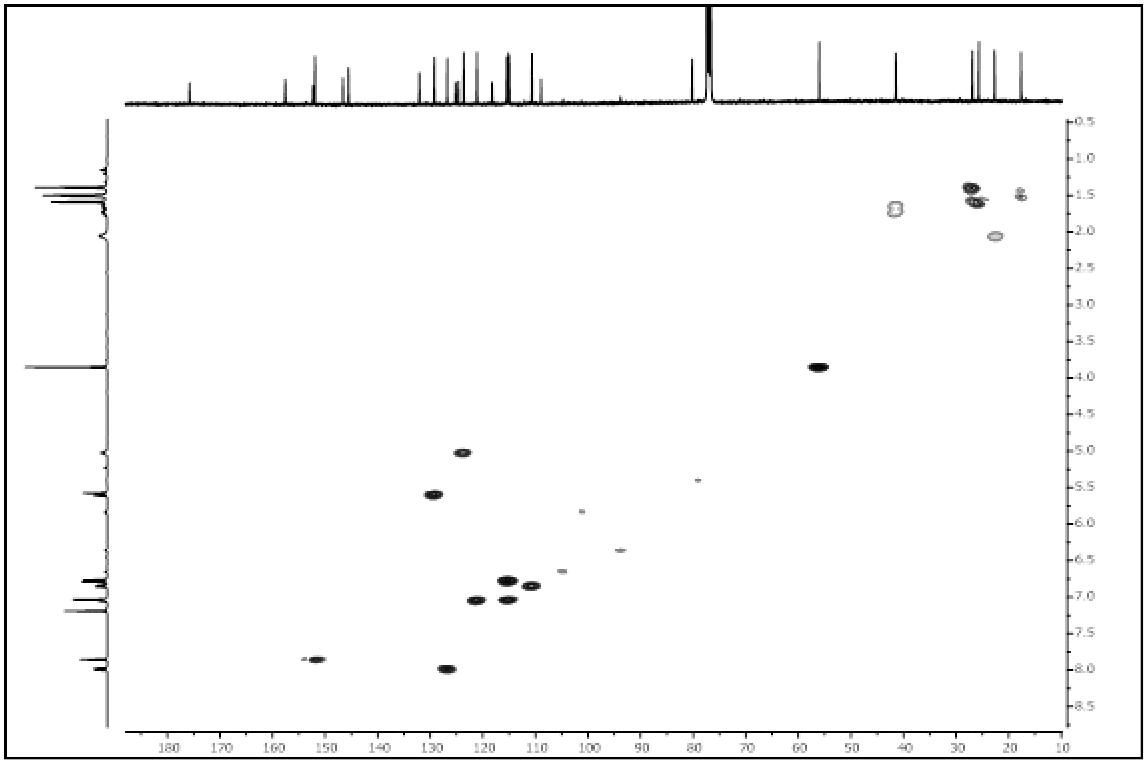

Figure S11. HMQC spectrum of dalpulapan B (**2**)

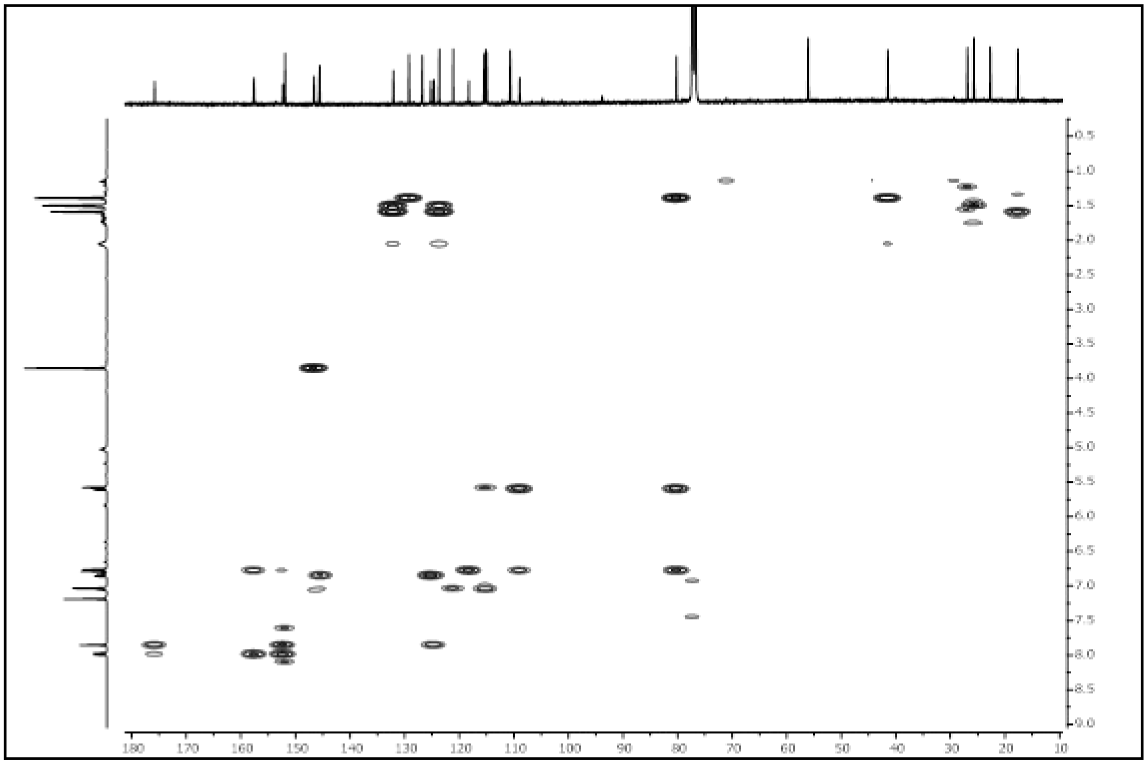

Figure S12. HMBC spectrum of dalpulapan B (**2**)

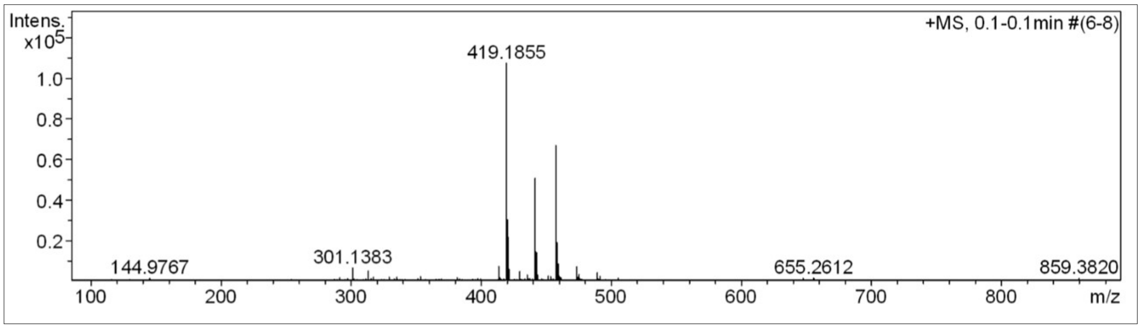

Figure S13. MS spectrum of dalpulapan B (**2**)

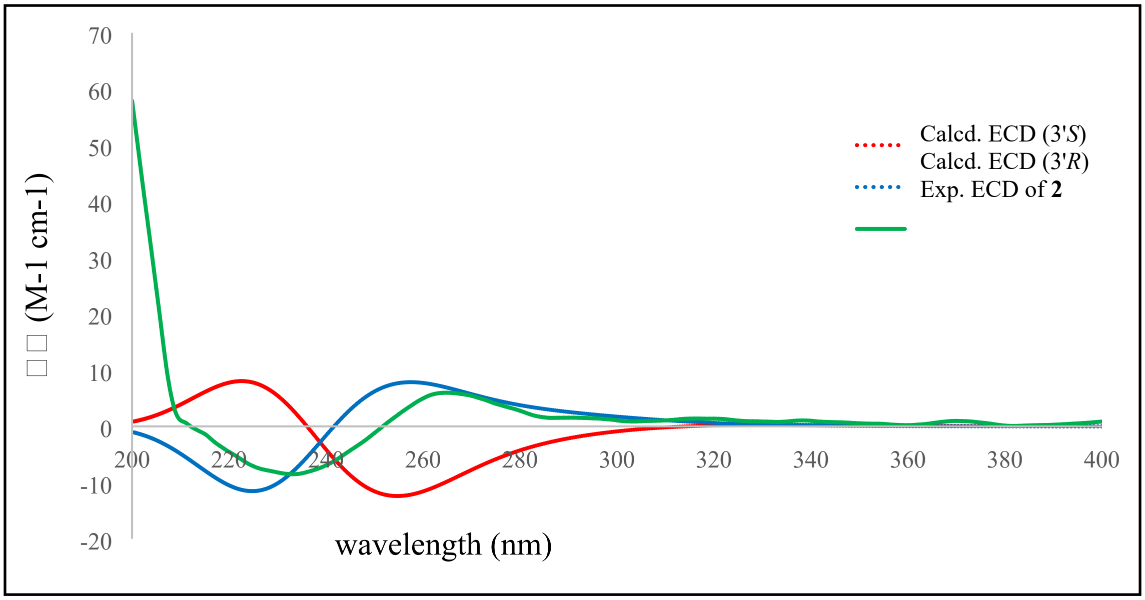

Figure S14. ECD spectrum of dalpulapan B (**2**)

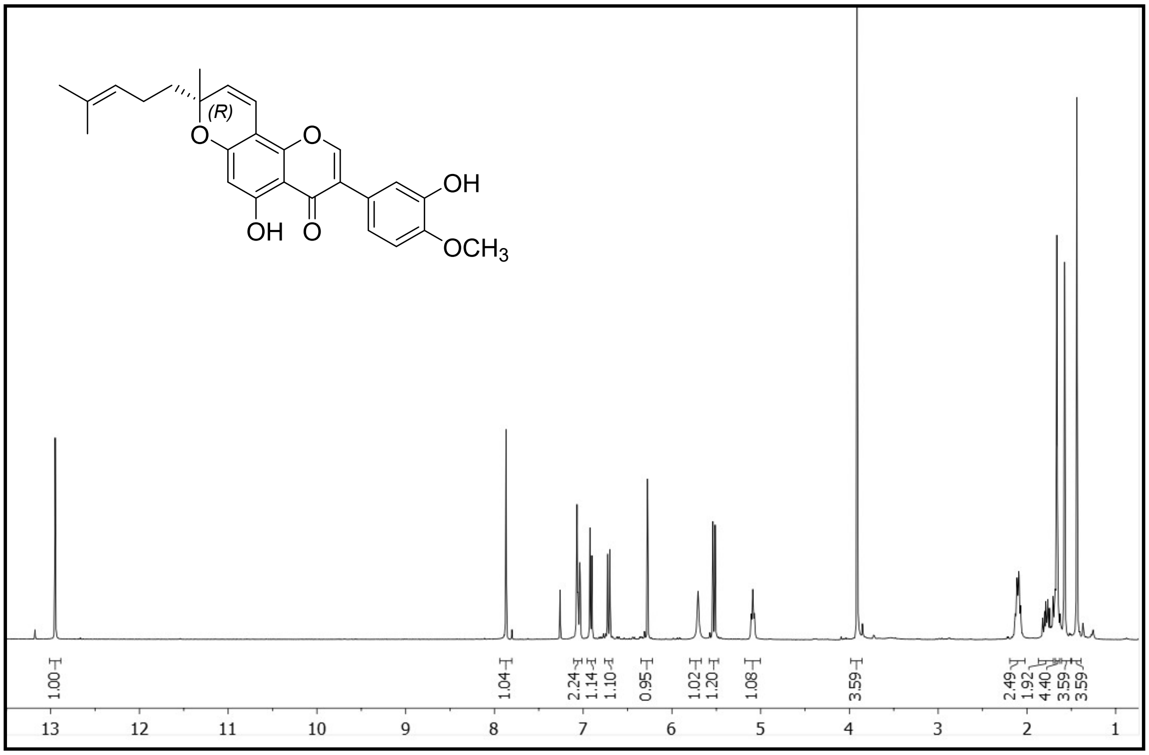

Figure S15. ^{1}H NMR spectrum of dalpulapan C (**3**)

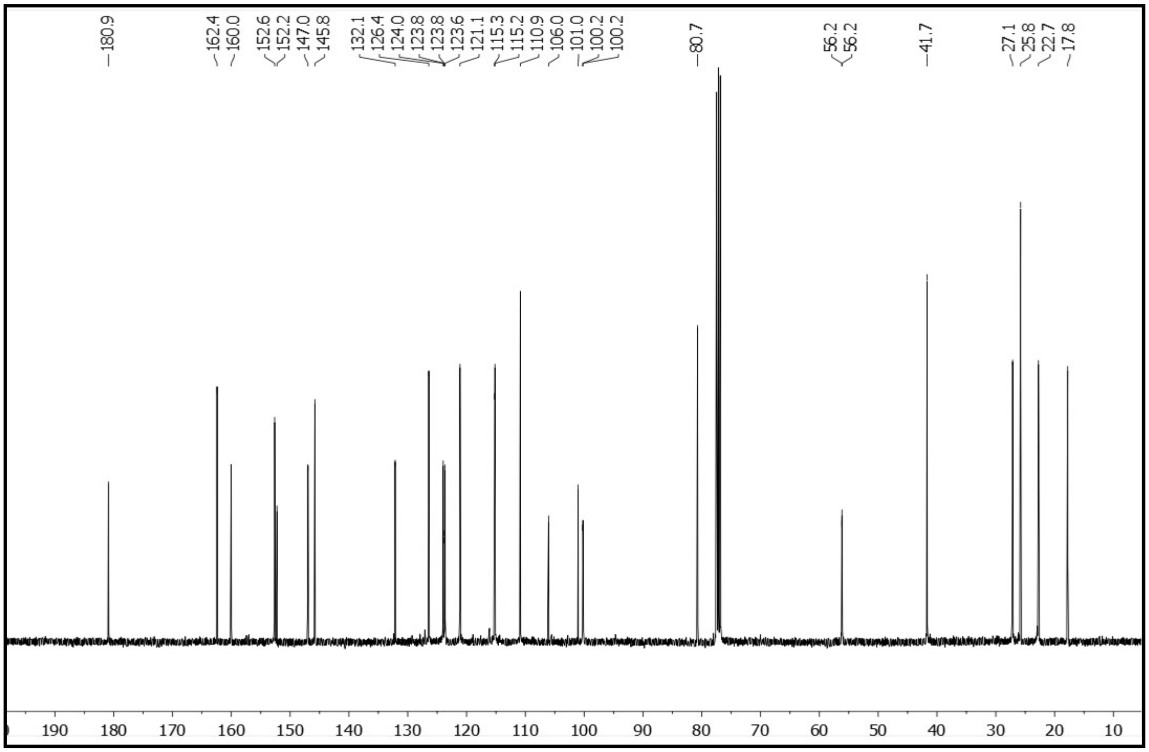

Figure S16. ^{13}C NMR spectrum of dalpulapan C (**3**)

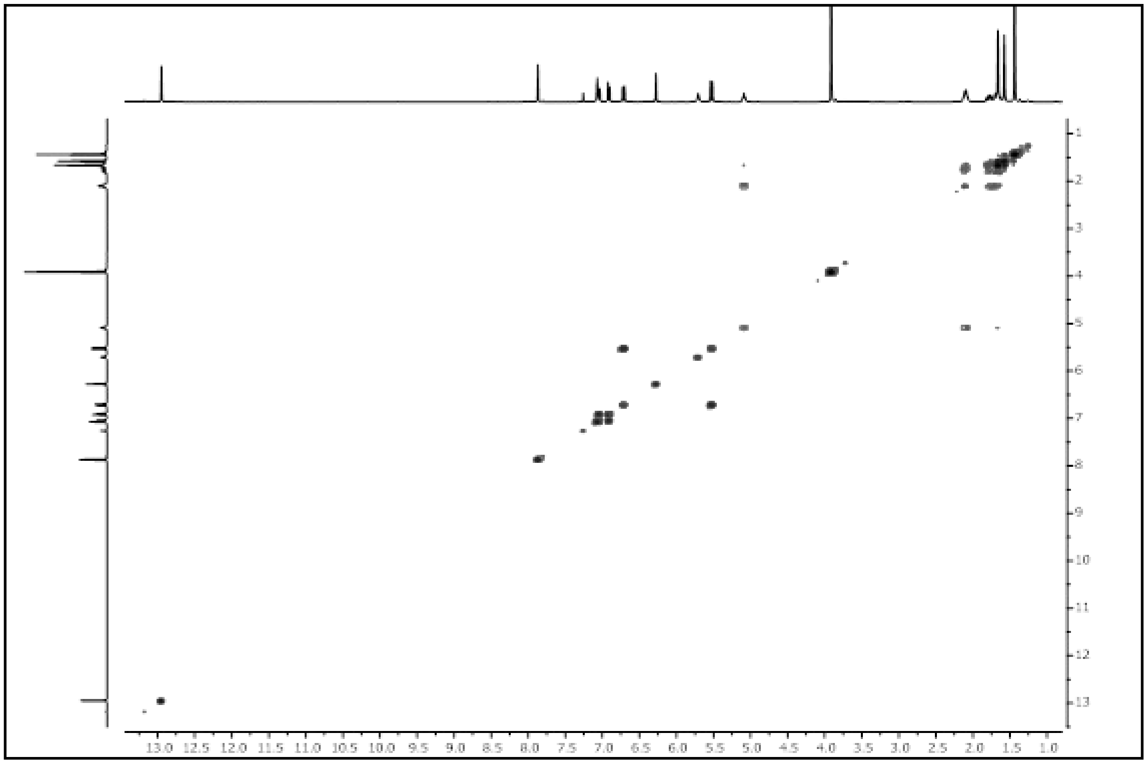

Figure S17. ^{1}H-^{1}H COSY spectrum of dalpulapan C (**3**)

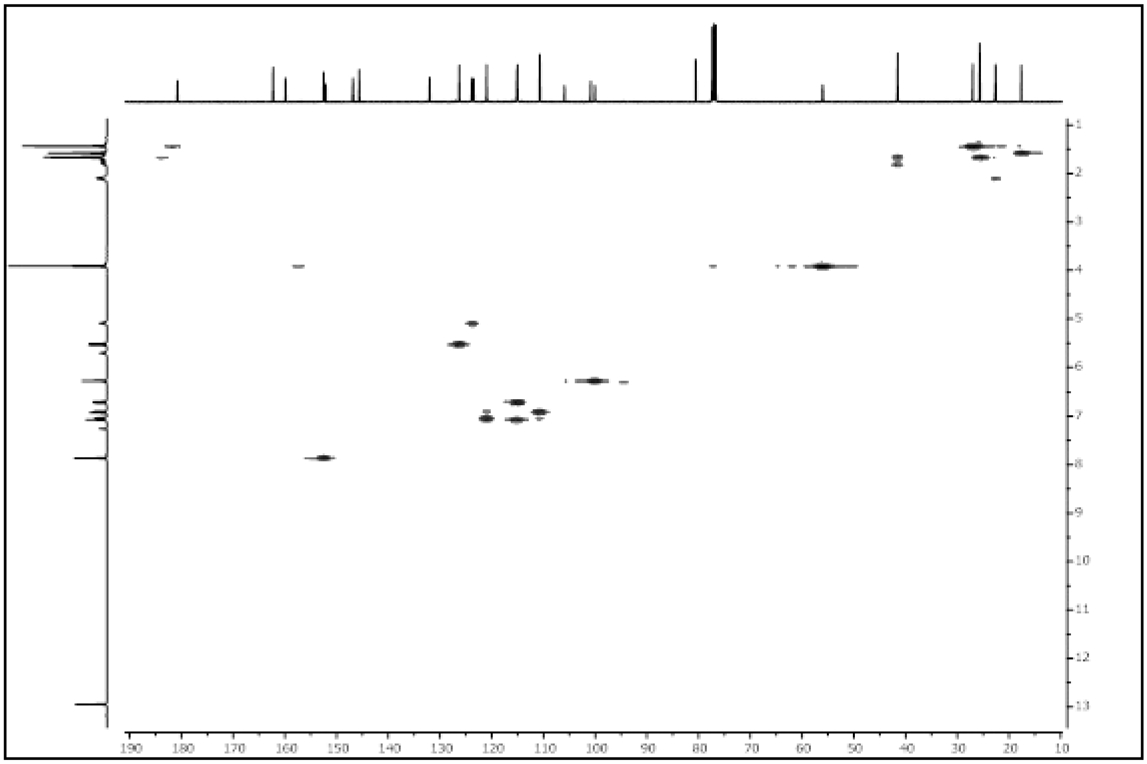

Figure S18. HMQC spectrum of dalpulapan C (**3**)

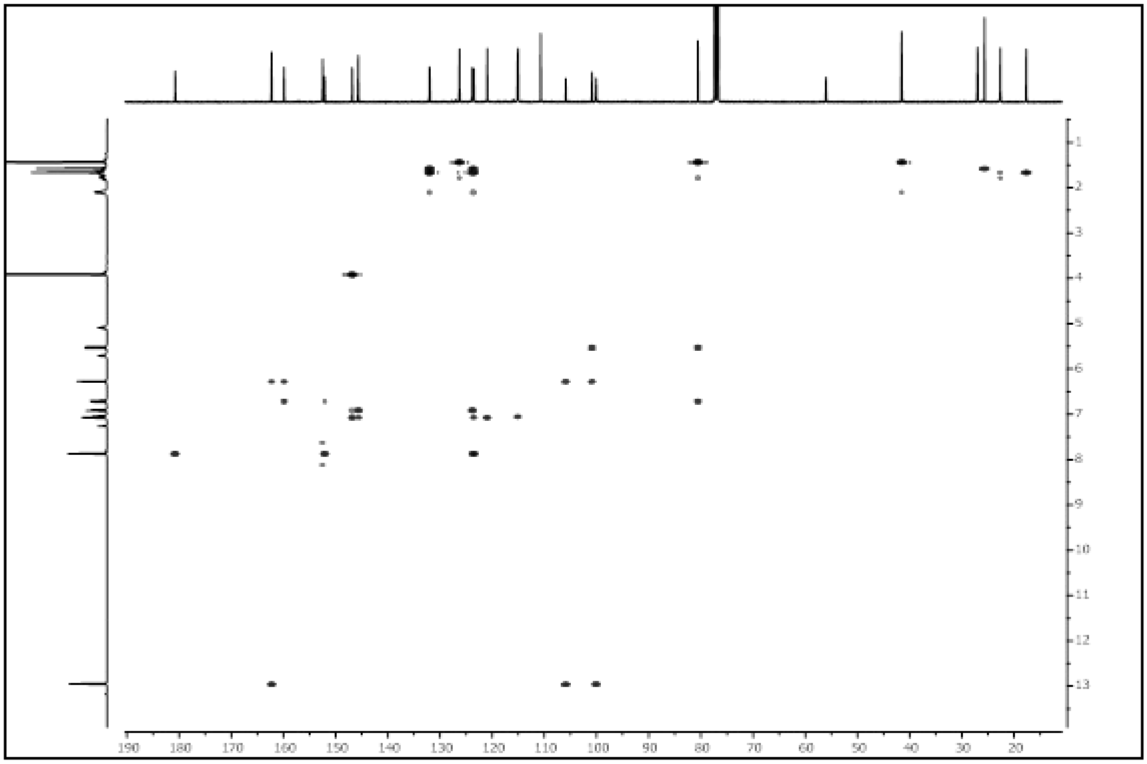

Figure S19. HMBC spectrum of dalpulapan C (**3**)

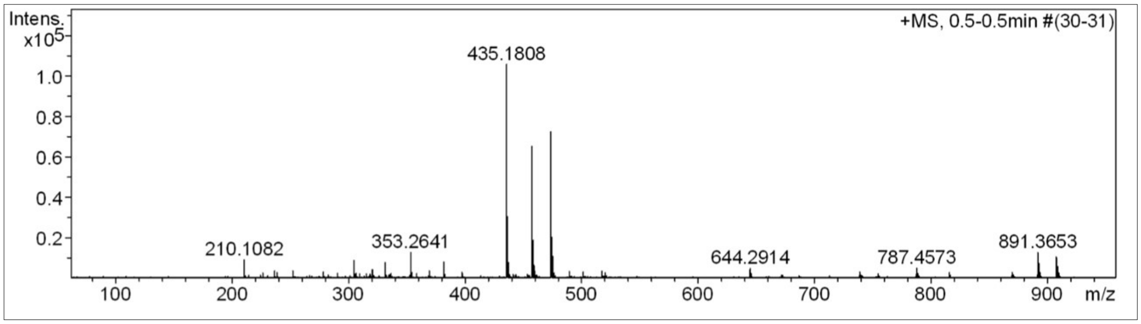

Figure S20. MS spectrum of dalpulapan C (**3**)

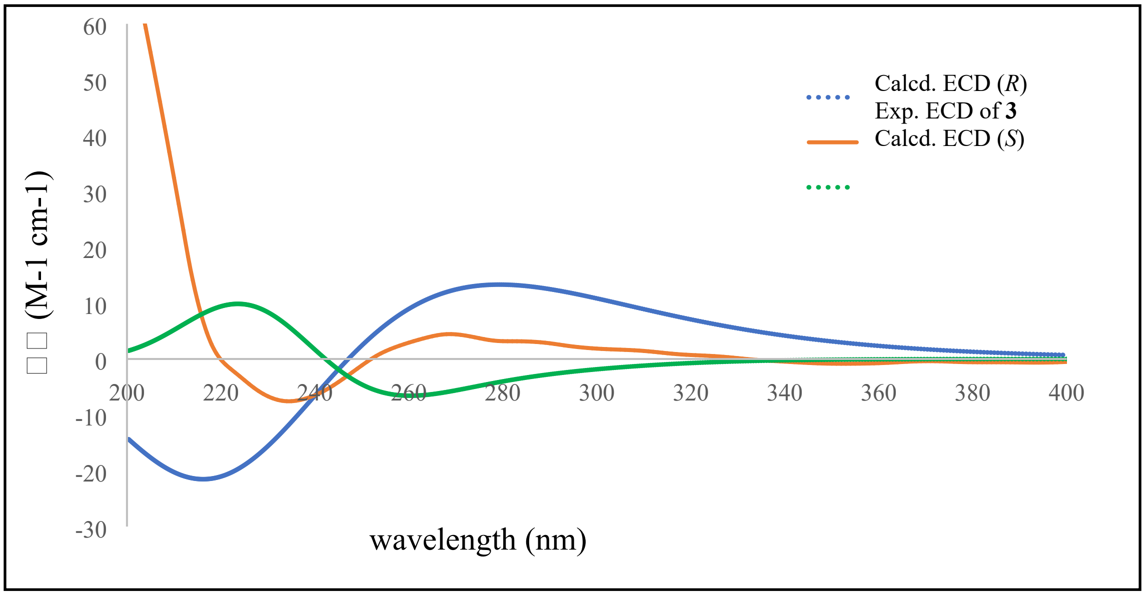

Figure S21. ECD spectrum of dalpulapan C (**3**)

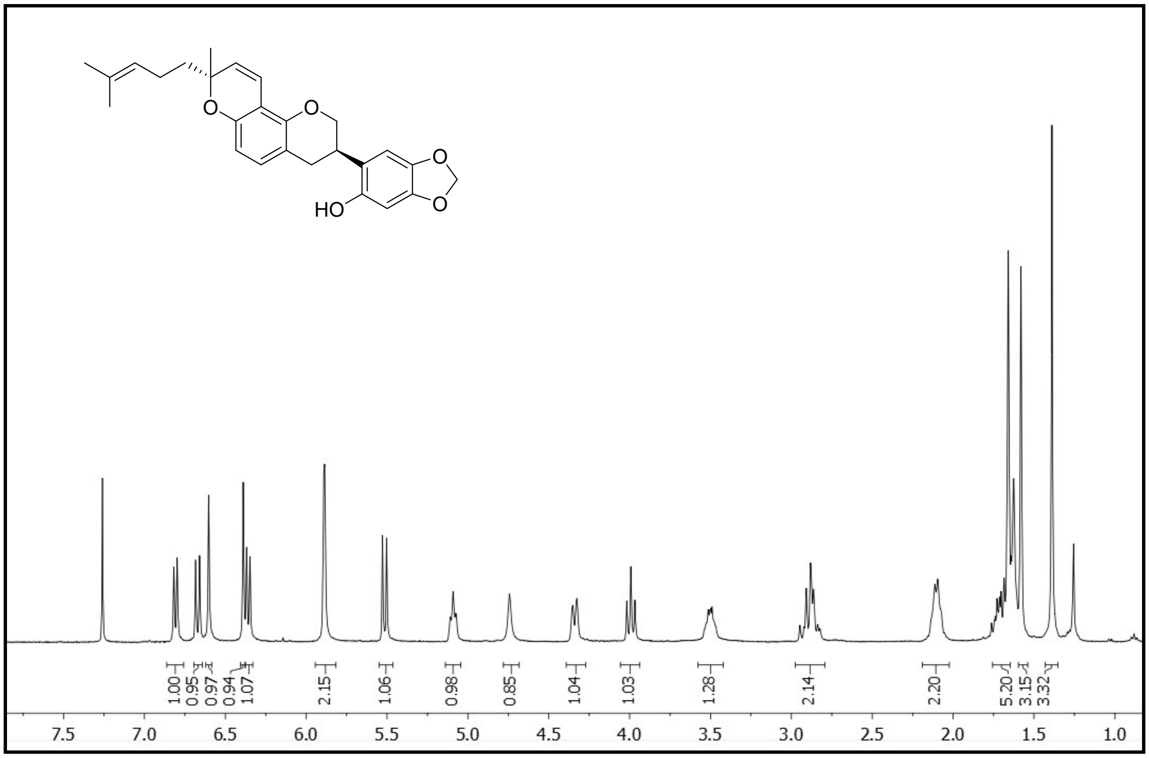

Figure S22. ^{1}H NMR spectrum of dalpulapan D (**4**)

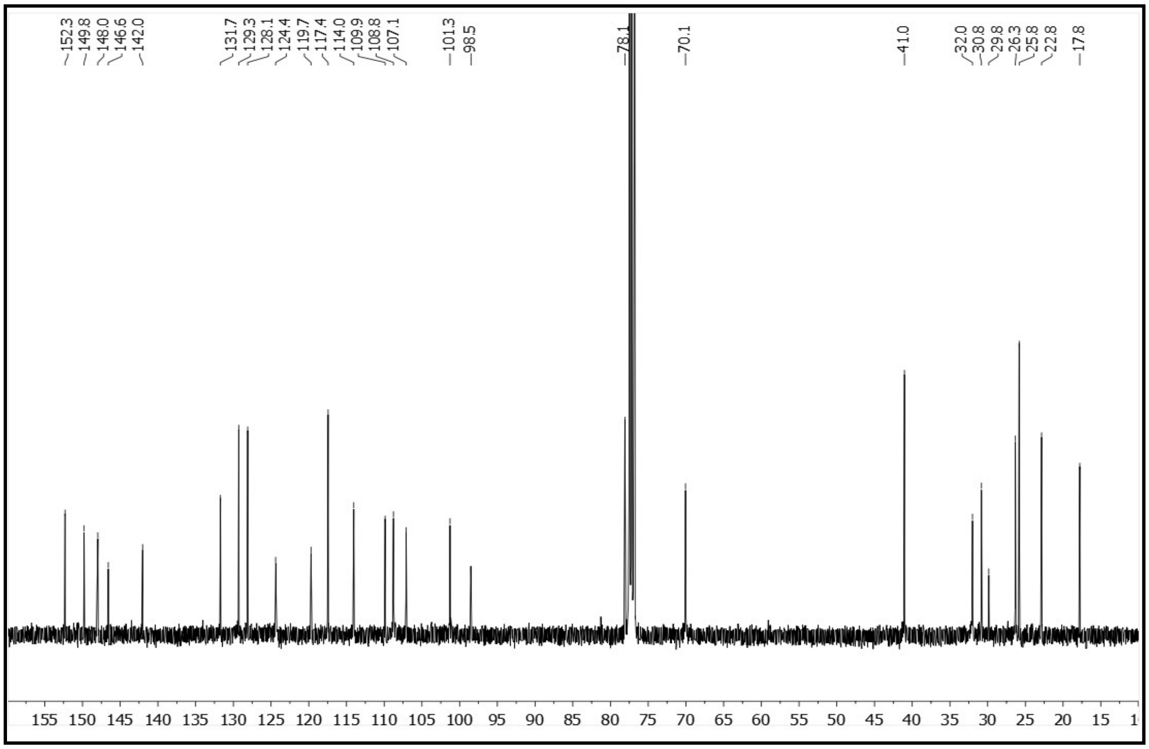

Figure S23. ^{13}C NMR spectrum of dalpulapan D (**4**)

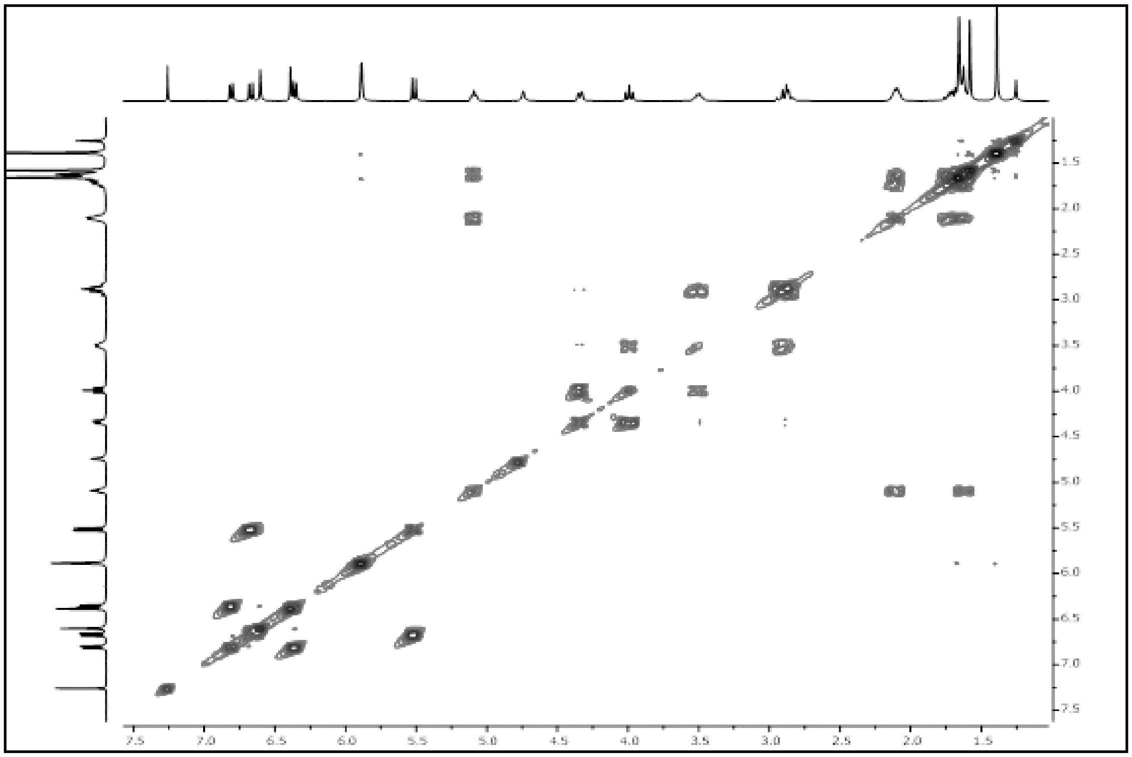

Figure S24. ^{1}H-^{1}H COSY spectrum of dalpulapan D (**4**)

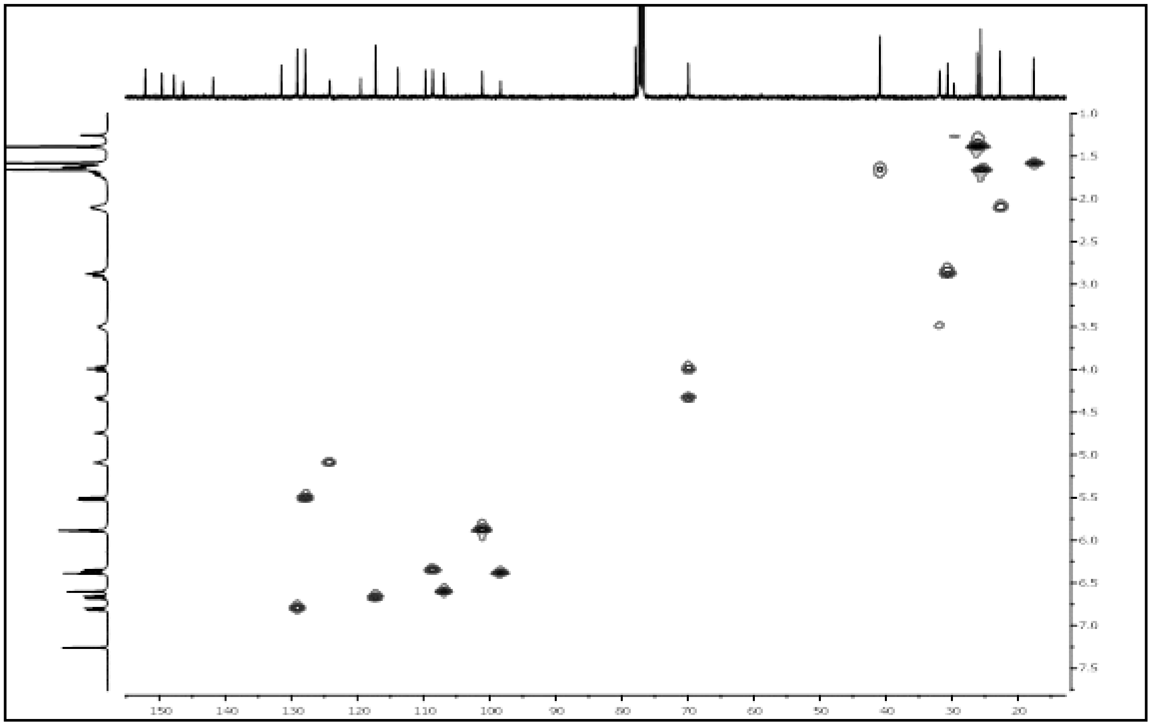

Figure S25. HMQC spectrum of dalpulapan D (**4**)

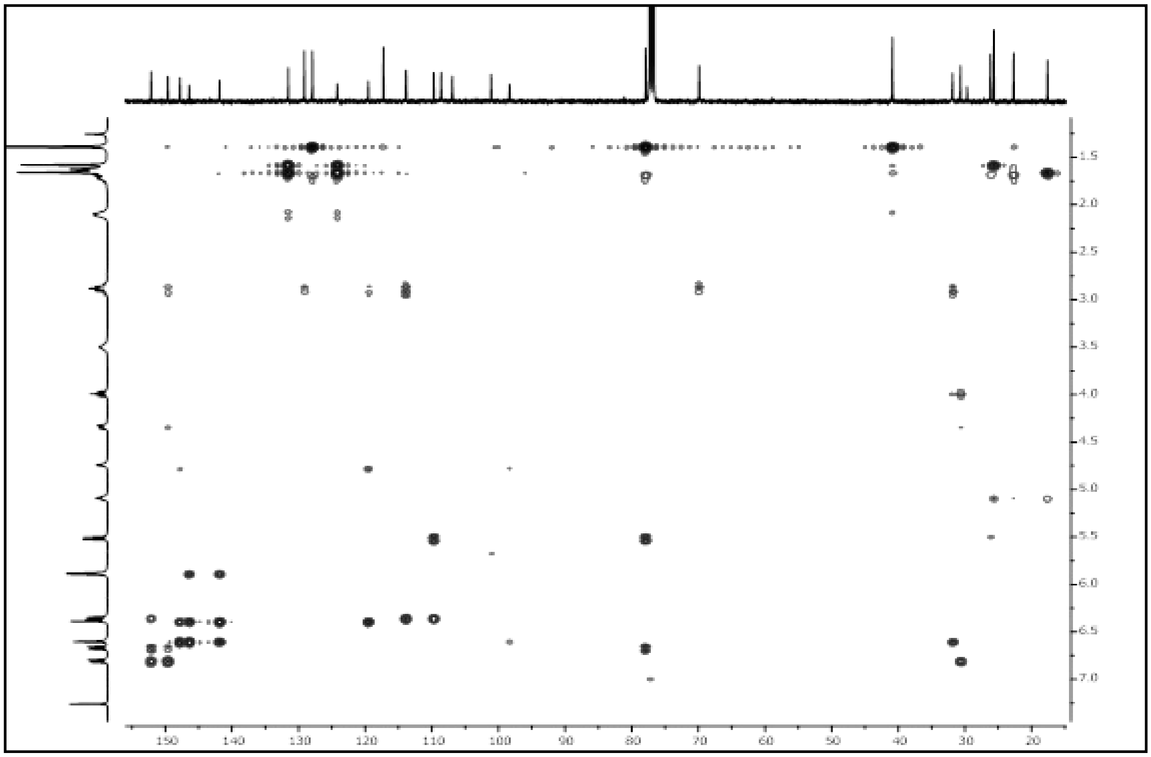

Figure S26. HMBC spectrum of dalpulapan D(**4**)

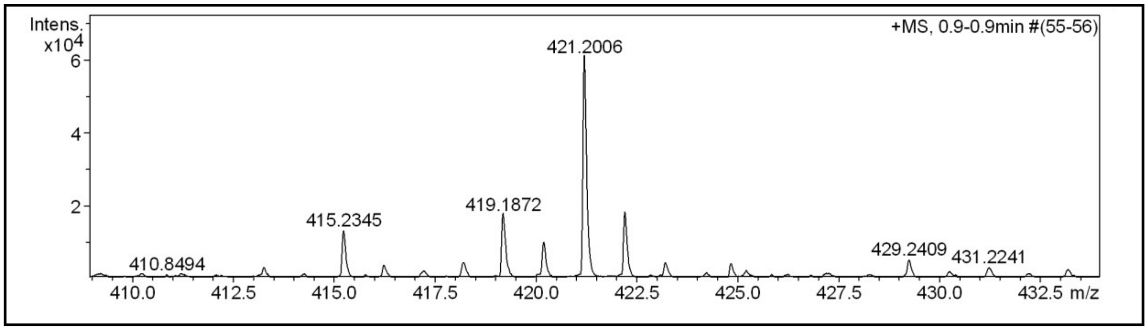

Figure S27. MS spectrum of dalpulapan D (**4**)

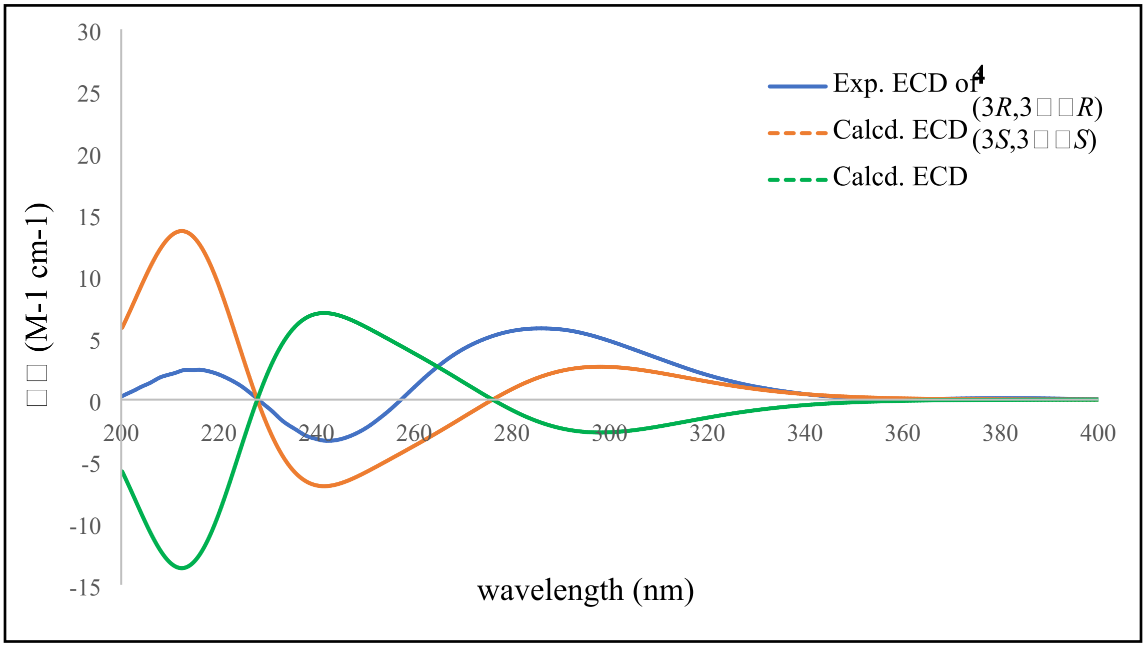

Figure S28. ECD spectrum of dalpulapan D (**4**)

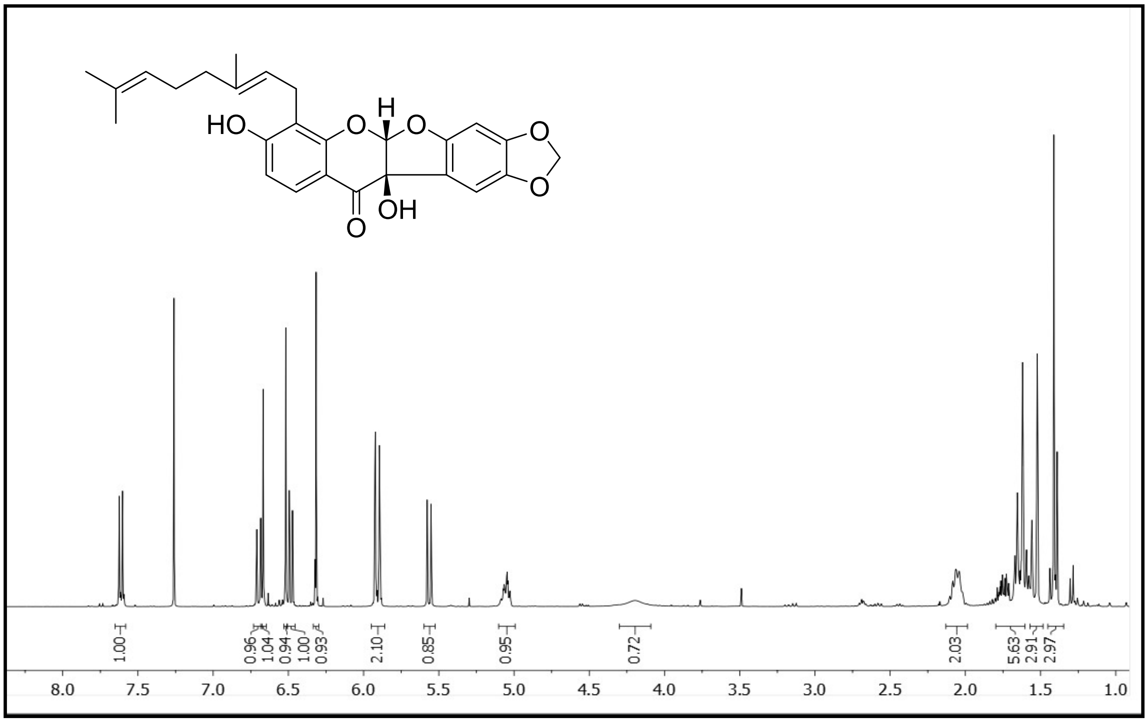

Figure S29. ^{1}H NMR spectrum of dalpulapan E (**5**)

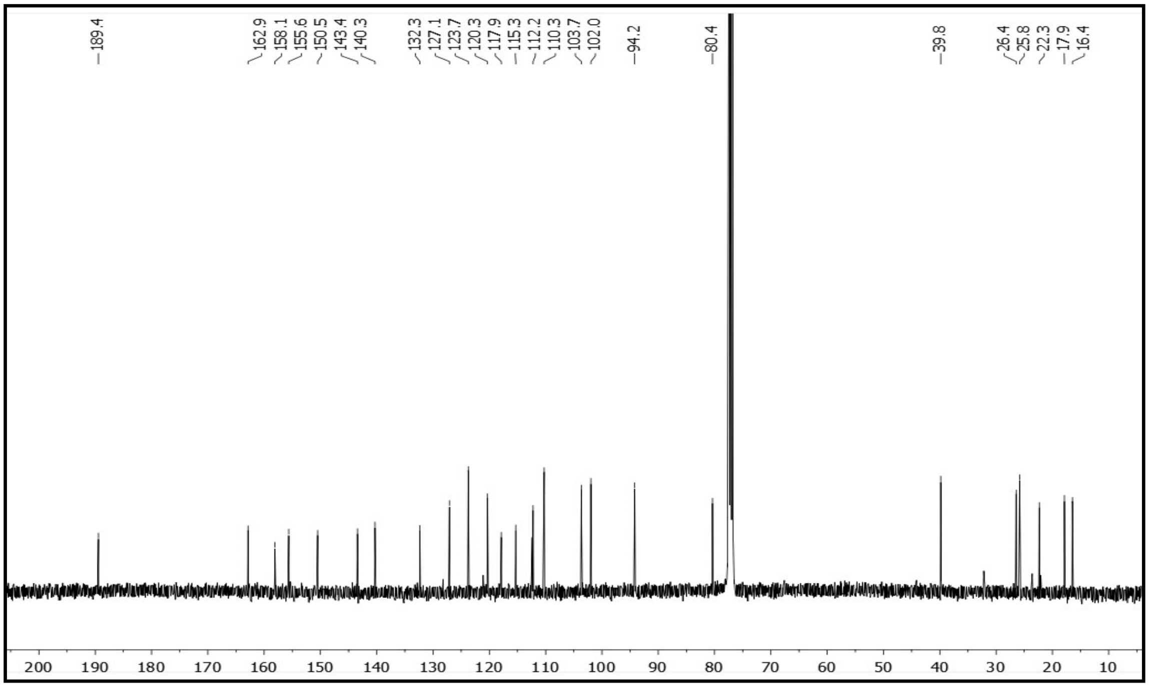

Figure S30. ^{13}C NMR spectrum of dalpulapan E (**5**)

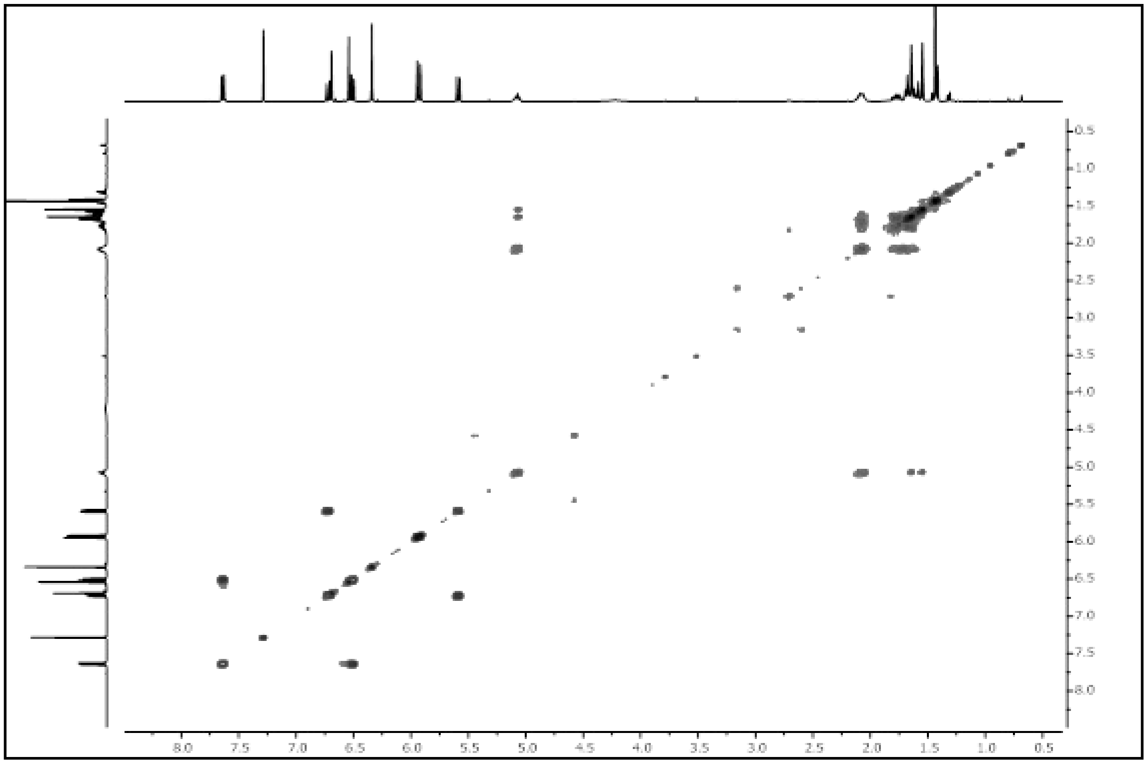

Figure S31. ^{1}H-^{1}H COSY spectrum of dalpulapan E (**5**)

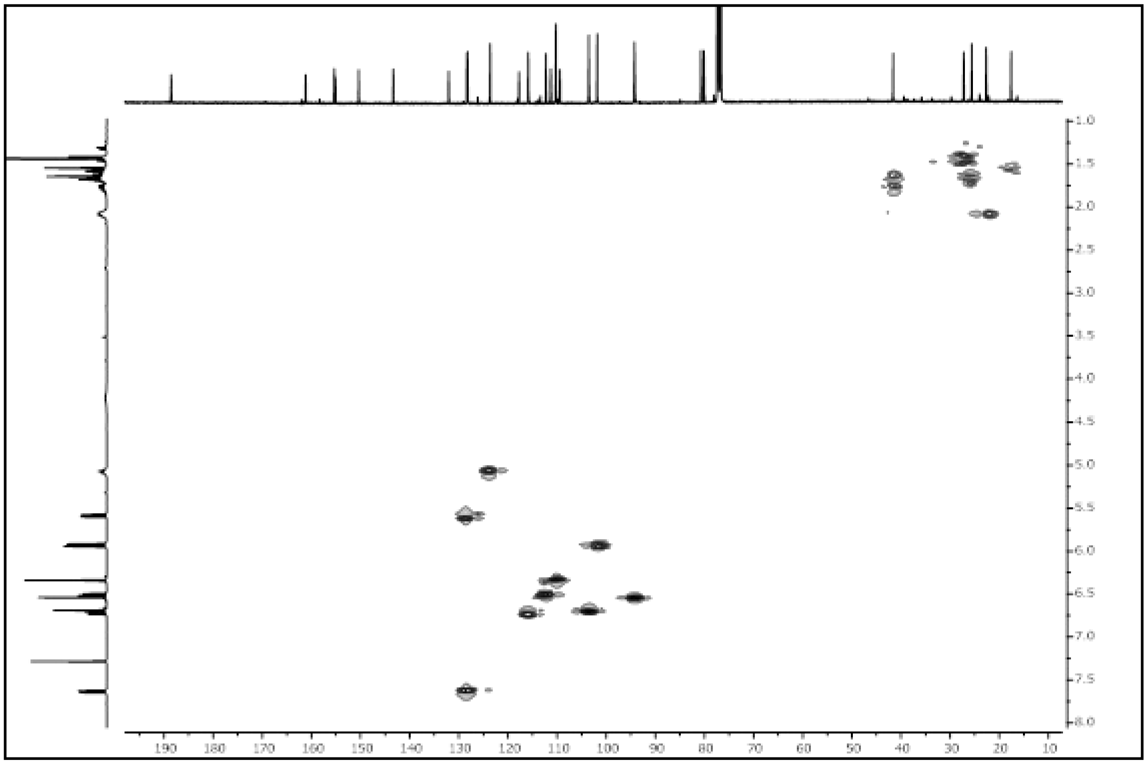

Figure S32. HMQC spectrum of dalpulapan E (**5**)

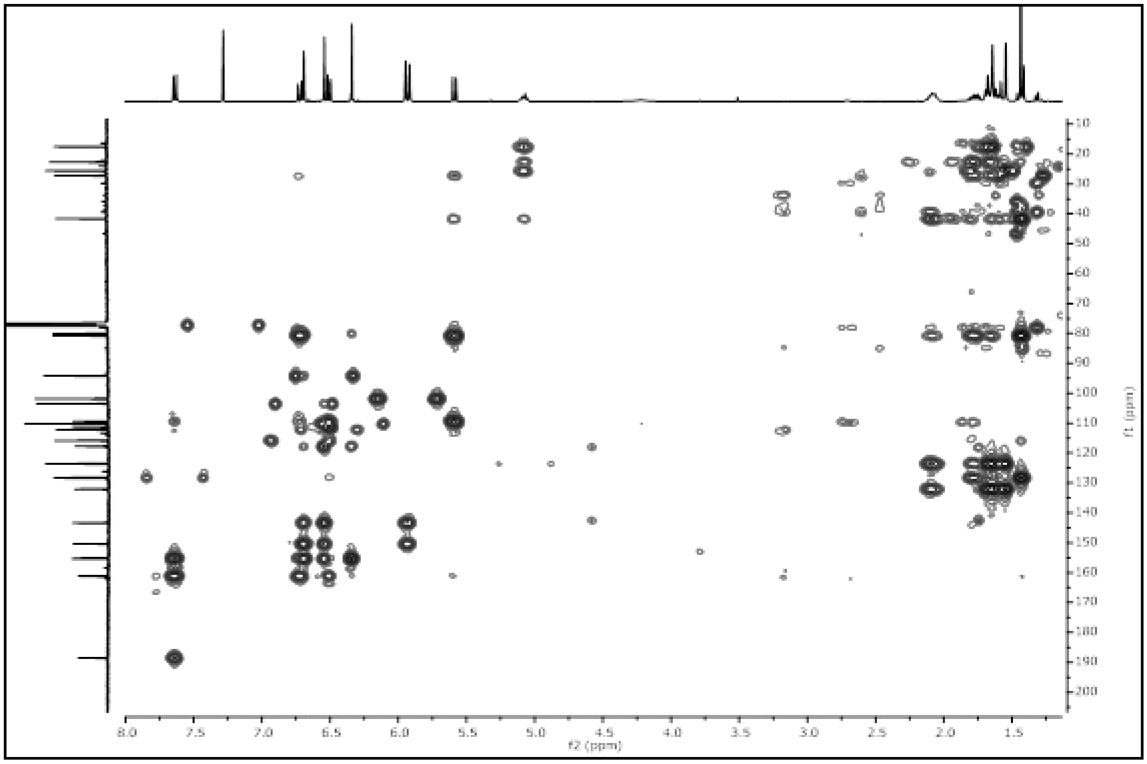

Figure S33. HMBC spectrum of dalpulapan E (**5**)

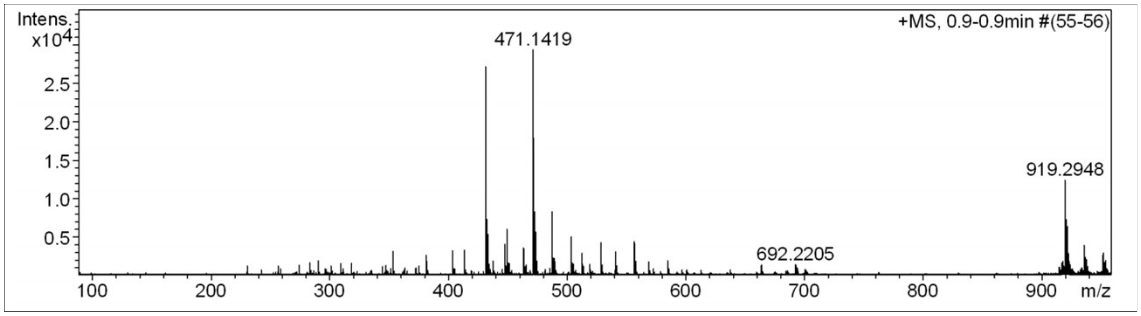

Figure S34. MS spectrum of dalpulapan E (**5**)

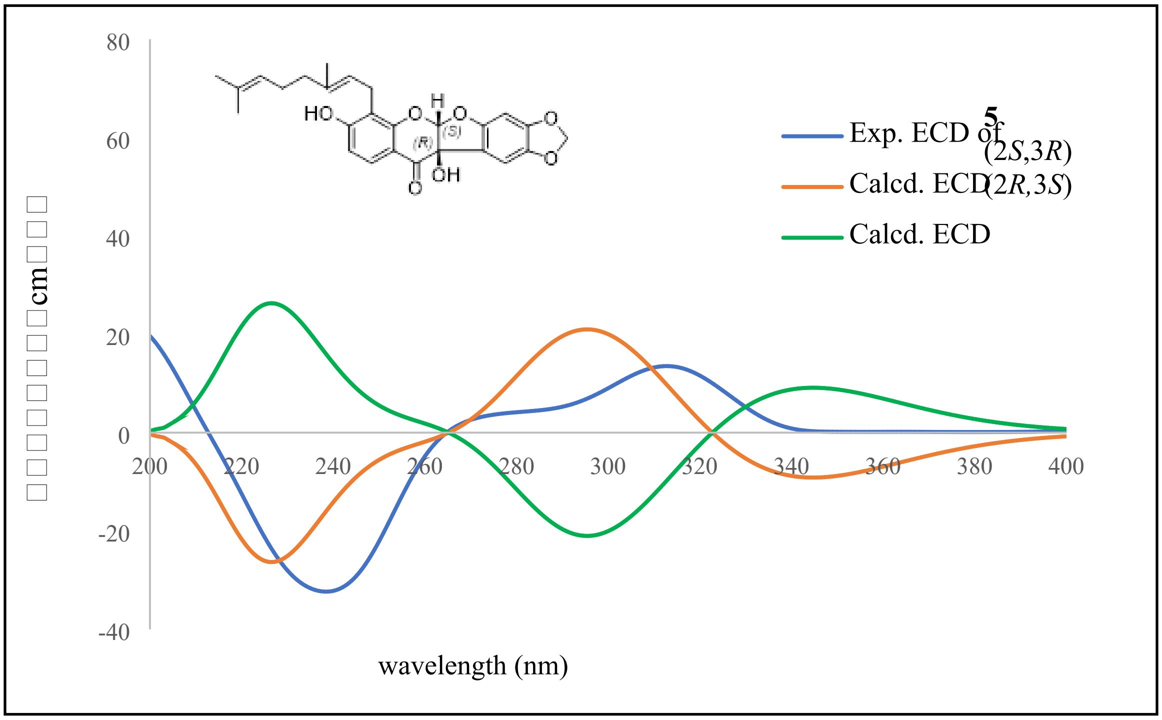

Figure S35. Experimental and calculated ECD spectrum of (2*S*,3*R*) dalpulapan E (**5**)

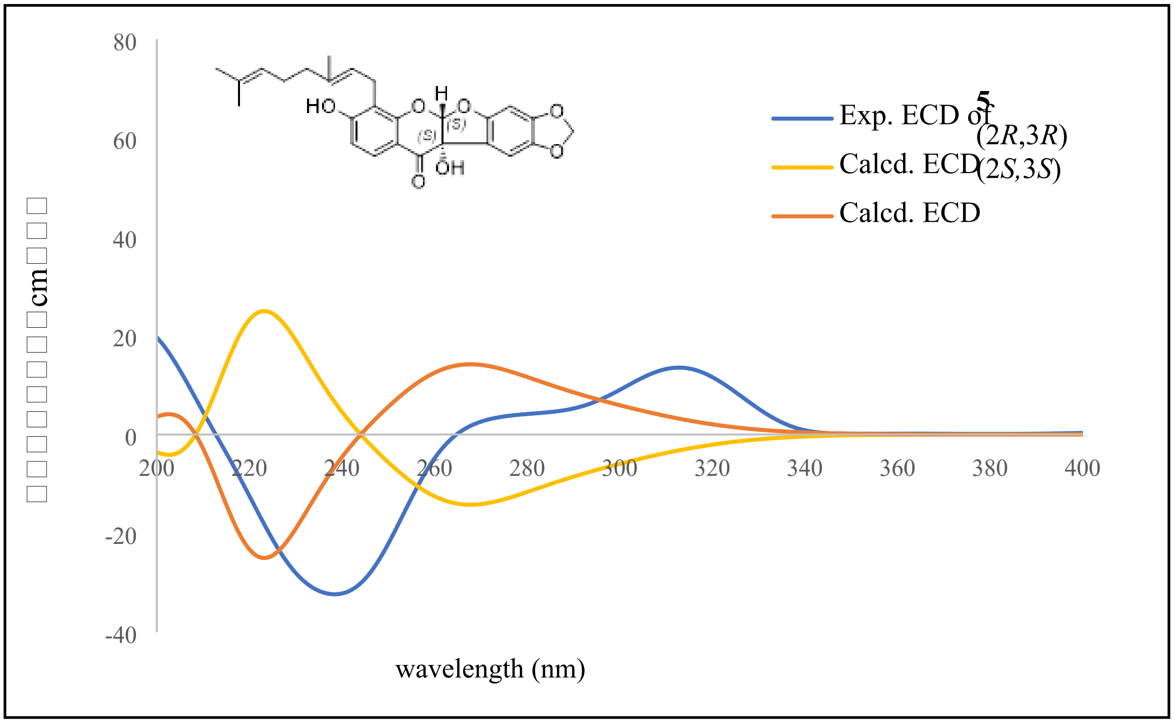

Figure S36. Calculated ECD spectrum of (2*R*,3*R* and 2*S*,3*S*) derivatives of **5**
